# Supplementary material for: An atypical atherogenic chemokine that promotes advanced atherosclerosis and hepatic lipogenesis
Source: Nat Commun. 2025 Mar 7;16:2297. doi: 10.1038/s41467-025-57540-z (PMC11889166; doi:10.1038/s41467-025-57540-z)
Supplement: Supplementary file 1 — Supplementary Information [file 41467_2025_57540_MOESM1_ESM.pdf]

Supplementary Information

An atypical atherogenic chemokine that promotes advanced atherosclerosis and hepatic lipogenesis

Omar El Bounkari[1,#,*], Chunfang Zan[1,#], Bishan Yang[1], Simon Ebert[1], Jonas Wagner[1], Elina Bugar[1], Naomi Kramer[1],Priscila Bourilhon[1], Christos Kontos[2], Marlies Zarwel[1], Dzmitry Sinitski[1], Jelena Milic[1], Yvonne Jansen[3], Wolfgang E. Kempf[4], Nadja Sachs[4,5], Lars Maegdefessel[4,5], Hao Ji[6], Ozgun Gokce[6,7,8,9], Fabien Riols[10], Mark Haid[10], Simona Gerra[1], Adrian Hoffmann[1,11], Markus Brandhofer[1], Maida Avdic[1], Richard Bucala[12], Remco T.A. Megens[3,5,13], Nienke Willemsen[3], Denise Messerer[14], Christian Schulz[14,15], Alexander Bartelt[3,5,16], Tobias Harm[17], Dominik Rath[17], Yvonne Döring[3,18], Meinrad Gawaz[17], Christian Weber[3,5,7,13], Aphrodite Kapurniotu[2], and Jürgen Bernhagen[1,5,7,*]

[1]Division of Vascular Biology, Institute for Stroke and Dementia Research (ISD), LMU Klinikum, Ludwig Maximilian University (LMU) Munich, 81377 Munich, Germany

[2]Division of Peptide Biochemistry, TUM School of Life Sciences, Technische Universität München (TUM), 85354 Freising, Germany

[3]Institute for Cardiovascular Prevention, LMU Klinikum, Ludwig Maximilian University (LMU) Munich, 80336 Munich, Germany

[4]Institute of Molecular Vascular Medicine, TUM Klinikum, Technische Universität München (TUM), 81675 Munich, Germany

[5]German Center for Cardiovascular Research (DZHK), partner site Munich Heart Alliance, 80802 Munich, Germany

[6]Systems Neuroscience Lab, Institute for Stroke and Dementia Research (ISD), LMU Klinikum, Ludwig Maximilian University (LMU) Munich, 81377 Munich, Germany

[7]Munich Cluster for Systems Neurology (SyNergy), 81377 Munich, Germany

[8]Department of Neurodegenerative Diseases and Geriatric Psychiatry, University Hospital Bonn, Venusberg-Campus 1, 53127 Bonn, Germany

[9]German Center for Neurodegenerative Diseases (DZNE) Bonn, 53127 Munich, Germany

[10]Metabolomics and Proteomics Core, Helmholtz Zentrum, 85764 Neuherberg, Germany

[11]Department of Anaesthesiology, LMU Klinikum, Ludwig Maximilian University (LMU) Munich, 81377 Munich, Germany

[12]Yale University School of Medicine, New Haven, CT 06520, USA

[13]Cardiovascular Research Institute Maastricht (CARIM), Maastricht University, 6229 Maastricht, The Netherlands

[14]Department of Medicine I, LMU Klinikum, Ludwig Maximilian University (LMU) Munich, 81377

Munich, Germany
[15]Department of Immunopharmacology, Mannheim Institute for Innate Immunoscience (MI3), Medical Faculty Mannheim, University of Heidelberg, 68167 Mannheim, Germany
[16]Institute for Diabetes and Cancer (IDC), Helmholtz Center Munich, German Research Center for Environmental Health, 85764 Neuherberg, Germany
[17]Department of Cardiology and Angiology, University Hospital Tübingen, Eberhard-Karls-University Tübingen, Tübingen, Germany
[18]Division of Angiology, Swiss Cardiovascular Center, Inselspital, Bern University Hospital, University of Bern, Bern, Switzerland

[#]These authors contributed equally
*Corresponding authors

*Correspondence:
Omar El Bounkari, PhD
Division of Vascular Biology
Institute for Stroke and Dementia Research (ISD)
LMU University Hospital (LMU Klinikum)
Ludwig-Maximilians-Universität (LMU) München
Feodor-Lynen-Straße 17, 81377 Munich, Germany
Tel.: 0049-89 4400 - 46151
E-Mail: Omar.El_Bounkari@med.uni-muenchen.de

Professor Jürgen Bernhagen, PhD
Chair of Vascular Biology
Institute for Stroke and Dementia Research (ISD)
LMU University Hospital (LMU Klinikum)
Ludwig-Maximilians-Universität (LMU) München
Feodor-Lynen-Straße 17, 81377 Munich, Germany
Tel.: 0049-89 4400 - 46151
E-Mail: juergen.bernhagen@med.uni-muenchen.de

Table of Contents

Supplementary Tables

Supplementary Table 1
Supplementary Table 2
Supplementary Table 3
Supplementary Table 4

Supplementary Figures

Supplementary Figure 1
Supplementary Figure 2
Supplementary Figure 3
Supplementary Figure 4
Supplementary Figure 5
Supplementary Figure 6
Supplementary Figure 7
Supplementary Figure 8
Supplementary Figure 9
Supplementary Figure 10
Supplementary Figure 11
Supplementary Figure 12
Supplementary Figure 13
Supplementary Figure 14
Supplementary Figure 15
Supplementary Figure 16
Supplementary Figure 17
Supplementary Figure 18
Supplementary Figure 19
Supplementary Figure 20
Supplementary Figure 21
Supplementary Figure 22
Supplementary Figure 23
Supplementary Figure 24
Supplementary Figure 25
Supplementary Figure 26
Supplementary Figure 27
Supplementary Figure 28
Supplementary Figure 29
Supplementary Figure 30
Supplementary Figure 31
Supplementary Figure 32

References cited in supplementary information

Supplementary Table 1. List of primers and sequences**.**

**Human primers**	**Sequence**
SREBP1 FWD	5’-ACGGCAGCCCCTGTAACGACCACTGTGA-3’
SREBP1 REV	5’-TGCCAAGATGGTTCCGCCACTCACCAGG-3’
SREBP2 FWD	5’-AGCTGGTCTGTGAAG-3’
SREBP2 REV	5’-CGCAATGGGGTCAGC-3’
FASN FWD	5’-GAAACTGCAGGAGCTGTC-3’
FASN REV	5’-CACGGAGTTGAGGCGCAT-3’
LDLR FWD	5’-GTGTCACAGCGGCG-3’
LDLR REV	5’-CGCACTCTTTGATG-3’
β-Actin FWD	5’-AGAGCTACGAGCTGCCTGAC-3’
β-Actin REV	5’-CGTGGATGCCACAGGACT-3’
**Murine primers**	**Sequence**
Srebp1 FWD	5’-GGCAAAGGAGGCACTACAG-3’
Srebp1 REV	5’-AGATACAGGATGCCAACAG-3’
Srebp2 FWD	5’-CCAAAGAAGGAGAGAGGCGG-3’
Srebp2 REV	5’-CGCCAGACTTGTGCATCTTG-3’
Fasn FWD	5'-CCGAGTCAGAGAACCTACAG-3´
Fasn REV	5'-CTTCCATCTCCTGTCATCAT-3´
Ldlr FWD	5’-ACCTGCCGACCTGATGAATTC-3’
Ldlr REV	5’-GCAGTCATGTTCACGGTCACA-3’
D-dt/Mif-2 FWD	5´-CCAGCTTCTTCAAGTTCCTCA-3´
D-dt/Mif-2 REV	5´-GGGAAGAAGCGGATAACGAT-3´
Pcsk9 FWD	5´-CACCTTTGGGTCGAGTGCTGAG-3´
Pcsk9 REV	5´-CGCTGTTGAAGTCGGTGATG-3´
Cd36 FWD	5'-GAACCACTGCTTTCAAAAACTGG-3´
Cd36 REV	5'-TGCTGTTCTTTGCCACGTCA-3´
Dgat1	5'-GTGTGTGGTGATGCTGATCC-3´
Dgat1 REV	5'-GATGCAATAATCACGCATGG-3´
Dgat2 FWD	5'-AGGCCCTATTTGGCTACGTT-3´
Dgat2 REV	5'-GATGCCTCCAGACATCAGGT-3´
Hmgcs1 FWD	5-GCCGTGAACTGGGTCGAA-3´
Hmgcs1 REV	5´-GCATATATAGCAATGTCTCCTGCAA-3´
Hmgcr FWD	5´-CTTGTGGAATGCCTTGTGATTG-3´
Hmgcr REV	5´-AGCCGAAGCAGCACATGAT-3´
Pparg FWD	5'-TGACCCAATGGTTGCTGATTACA-3´
Pparg REV	5'-CAATGGCCATGAGGGAGTTAGA-3´
Ppara FWD	5´-CCCTGCCATTGTTAAGACC-3´
Ppara REV	5´-TGCTGCTGTTCCTGTTTTC-3´
Mogat1 FWD	5´-GAGTAACGGGCCGGTTTC-3´
Mogat1 REV	5´-AGACATTGCCACCTCCATCCT-3´
Cpt1 FWD	5'-AGACTTCCAACGCATGACAGCACTG-3´
Cpt1 REV	5'-CTCGGCCCCGCAGGTAGATG-3´
Acox-1 FWD	5'-CAAGACCCAAGAGTTCATT-3´
Acox-1 REV	5'-TTCAGGTAGCCATTATCCA-3´
Cpt2 FWD	5´-GGCCACCAACTTGACTGTTT-3´
Cpt2 REV	5´-GAAGGAACAAAGCGGATGAG´-3´
Pgc1 FWD	5´-CCCTGCCATTGTTAAGACC-3´
Pgc1 REV	5´-TGCTGCTGTTCCTGTTTTC-3´

Akt2 FWD	5´-GAATGCCAGCTGATGAAGACTGA-3´
Akt2 REV	5´-CTACATGGAAGGTCCTCTCGATGA-3´
Cyp7a1 FWD	5´-AGCAACTAAACAACCTGCCAGTACTA-3´
Cyp7a1 REV	5´-GTCCGGATATTCAAGGATGCA-3´
Cyp8b1 FWD	5´- ACGCTTCCTCTATCGCCTGAA-3´
Cyp8b1 REV	5´- GTGCCTCAGACGCAGAGGAT-3´
Cyp27a1 FWD	5´-TGGGGTAGACACGACATCCA-3´
Cyp27a1 REV	5´-CTTCCCGAAGGGTACCACAC-3
Cyp7b1 FWD	5´-GCTATGGAAGTCCTGCGTGA´-3´
Cyp7b1 REV	5´-AATAGCGCTTTCCAGGCAGA
ApoA4 FWD	5´-CAGCTGACCCCATACATCCAG-3´
ApoA4 REV	5´-TCATCGAGGTGTGCAGGTTG-3´
Ces2a FWD	5´-CTCACAGCCGGCCATGT-3´
Ces2a REV	5´-AGATTCATTTCCTTCGCATCCT´-3´
Ces2e FWD	5´-CTTGTCTTTGGCTACCAGTTCG-3´
Ces2e REV	5´-TTGCTCCTCTTCCTCAGTGTAAGG-3´
Acat-1 FWD	5´-AGCCCAGAAAAATTTCATGGACACATACAG-3
Acat-1 REV	5´-CCCTTGTTCTGGAGGTGCTCTCAGATCTTT´-3
Acc FWD	5'-TGAGGAGGACCGCATTTATC-3´
Acc REV	5'-GCATGGAATGGCAGTAAGGT-3´
S1p FWD	5´-TGCTCCCACCTGACTTTGAAG-3´
S1p REV	5´-GCTGTGAAGTATCCGTTGAAAGC-3´
Sr-b1 FWD	5'-TTTGGAGTGGTAGTAAAAAGGGC-3´
Sr-b1 REV	5'-TGACATCAGGGACTCAGAGTAG-3´
Scd1 FWD	5'-GCGATACACTCTGGTGCTCA-3´
Scd1 REV	5'-CCCAGGGAAACCAGGATATT-3´
Scap FWD	5´-ATTTGCTCACCGTGGAGATGTT-3´
Scap REV	5´-GAAGTCATCCAGGCCACTACTAATG-3´
β-Actin FWD	5’-GGAGGGGGTTGAGGTGTT-3’
β-Actin REV	5’-GTGTGCACTTTTATTGGTCTCAA-3’

FWD: Forward, REV: Reverse

Supplementary Table 2. Blood cell count, body weight and serum lipid levels from female *Apoe*$^{-/-}$ mice *Mif-2*$^{-/-}$*Apoe*$^{-/-}$ mice on cholesterol-rich high-fat diet (HFD) for 4.5 weeks and 12 weeks (n=8 mice). Values are expressed as means ± SD and statistically analyzed using an unpaired two-tailed t-test.

**Female, 4.5-week HFD**	***Apoe***$^{-/-}$	***Mif-2***$^{-/-}$***Apoe***$^{-/-}$	***P*** **value**
**Serum lipid levels**			
Triglycerides (mg/dL)	188.14 ± 12.24	160.3 ± 36.36	**0.0197**
Cholesterol (mg/dL)	887.8 ± 104.49	703.9 ± 138.3	**0.0013**
**Blood cell percentages (%)**			
Lymphocytes (%)	59.11 ± 1.93	63.34 ± 2.33	**0.0313**
T lymphocytes (%)	17.71 ± 4.35	26.36 ± 5.05	**0.0409**
B-lymphocytes (%)	41.39 ± 5.16	36.94 ± 4.55	0.2437
Neutrophils (%)	22.28 ± 4.95	24.21 ± 4.3	0.5777
Monocytes (%)	3.71 ± 1.13	1.49 ± 0.52	**0.0118**
**Body weight**			
Weight (g)	24.42 ± 3.49	20.89 ± 1.25	**0.0222**
**Female, 12-week HFD**	***Apoe***$^{-/-}$	***Mif-2***$^{-/-}$***Apoe***$^{-/-}$	***P*** **value**
**Serum lipid levels**			
Triglycerides (mg/dL)	189.34 ± 8.91	180.38 ± 7.12	**0.0197**
Cholesterol (mg/dL)	1002.31 ± 113.94	850.56 ± 92.84	**0.0013**
**Blood cell percentages (%)**			
Lymphocytes (%)	69.46 ± 8.88	73.23 ± 7.89	0.3618
T lymphocytes (%)	24.52 ± 10.9	25.97 ± 3.70	0.7248
B-lymphocytes (%)	44.77 ± 5.47	47.17 ± 6.49	0.4061
Neutrophils (%)	13.19 ± 6.99	17.68 ± 6.49	0.1811
Monocytes (%)	3.48 ± 2.35	2.63 ± 0.70	0.3368
**Body weight**			
Weight (g)	29.75 ± 2.91	25.78 ± 3.2	**0.0140**

Shown are means ± SD. *P* values calculated by Student's t-test. Leukocytes were identified as CD45+; T lymphocytes as CD45+CD3+; B lymphocytes as CD45+CD19+; neutrophils as CD45+CD11b+Ly6G+; monocytes as CD45+CD11b+Ly6C+.

Supplementary Table 3. Baseline characteristics of CAD patients stratified according to chronic coronary symptom (CCS) and acute coronary syndrome (ACS). Continuous variables were compared using unpaired t-test (unpaired two-sided *P* values).

	**CCS (n=85)**	**ACS (n=47)**	***P* value**
Age (mean±SD)	70.3 (±9.4)	67.0 (±12.6)	0.120
Gender (male)	61 (71.8%)	35 (74.5%)	0.738
LVEF% (mean±SD)	53.1 (±10.1)	46.3 (±11.5)	**<0.001**
**CVRF (% of total)**			
Arterial hypertension	79 (92.9%)	37 (78.7%)	**0.017**
Hyperlipidemia	47 (55.3%)	21 (44.7%)	0.261
Diabetes	24 (28.2%)	10 (21.3%)	0.397
Active smoking	10 (11.8%)	12 (25.5%)	**0.039**
**Laboratory values (mean ± SD)**			
Leukocytes (1/µl)	7731 (±2155)	10733 (±4579)	**<0.001**
Platelets (*1000/µl)	247 (±60.4)	255 (±59.8)	0.460
CRP (mg/dl)	0.6 (±1.9)	2.1 (±4.1)	**0.032**
GFR (ml/min)	71.3 (±24.4)	76.1 (±29.0)	0.320
Creatinine (mg/dl)	1.1 (±0.6)	1.1 (±0.9)	1
Troponin I (µg/l)	0.01 (±0.03)	24.77 (±51.63)	**0.002**
CK (u/l)	104.1 (±60.5)	947.3 (±1655.7)	**0.002**
Total cholesterol (mg/dl)	172.4 (±40.8)	180.4 (±52.4)	0.426
HDL (mg/dl)	46.7 (±15.0)	39.7 (±10.6)	**0.028**
LDL (mg/dl)	92.6 (±31.6)	107.8 (±39.1)	**0.027**
Triglycerides (mg/dl)	150.5 (±69.1)	153.1(±105.9)	0.444
**Medication at admission (% of total)**			
ASA	53 (62.4%)	15 (31.9%)	**0.025**
P2Y12 inhibitors	31 (36.5%)	3 (6.4%)	**0.001**
ACE inhibitors	45 (52.9%)	15 (31.9%)	0.239
AT1 blockers	13 (15.3%)	4 (8.5%)	0.548
Beta blockers	58 (68.2%)	16 (34.0%)	**0.007**
Calcium channel blockers	22 (25.9%)	9 (19.1%)	0.931
Thiazide diuretics	26 (30.6%)	10 (21.3%)	0.766
Loop diuretics	17 (20.0%)	5 (10.6%)	0.427
Potassium sparing diuretics	16 (18.8%)	3 (6.4%)	0.146
Statins	49 (57.6%)	13 (27.7%)	**0.022**

LVEF, left ventricular ejection fraction; CVRF, cardiovascular risk factors; CRP, C-reactive protein; GFR, glomerular filtration rate; CK, creatine kinase; HDL, high density lipoprotein; LDL, low density lipoprotein; ASA, acetylsalicylic acid; P2Y12, adenosine diphosphate receptor P2Y12 inhibitors; ACE, angiotensin-converting enzyme; AT1, type I angiotensin.

Supplementary Table 4. Linear regression analysis with forward variable selection.

**After forward variable selection***	**Regression coefficient (95% CI)**	***P* value**
P2Y12 inhibitors	-0.37 (-0.59- -0.14)	**0.002**
Leukocytes	0.0 (0.0-0.0)	**0.027**
MIF-2	0.27 (0.07-0.47)	**0.010**

*Variables included in the model: arterial hypertension, active smoking, leucocytes, CRP, troponin I, CK, acetylsalicylic acid (ASA), adenosine diphosphate receptor $P2Y_{12}$ (P2Y12) inhibitors, beta blockers, statins, and MIF-2 (D-DT).

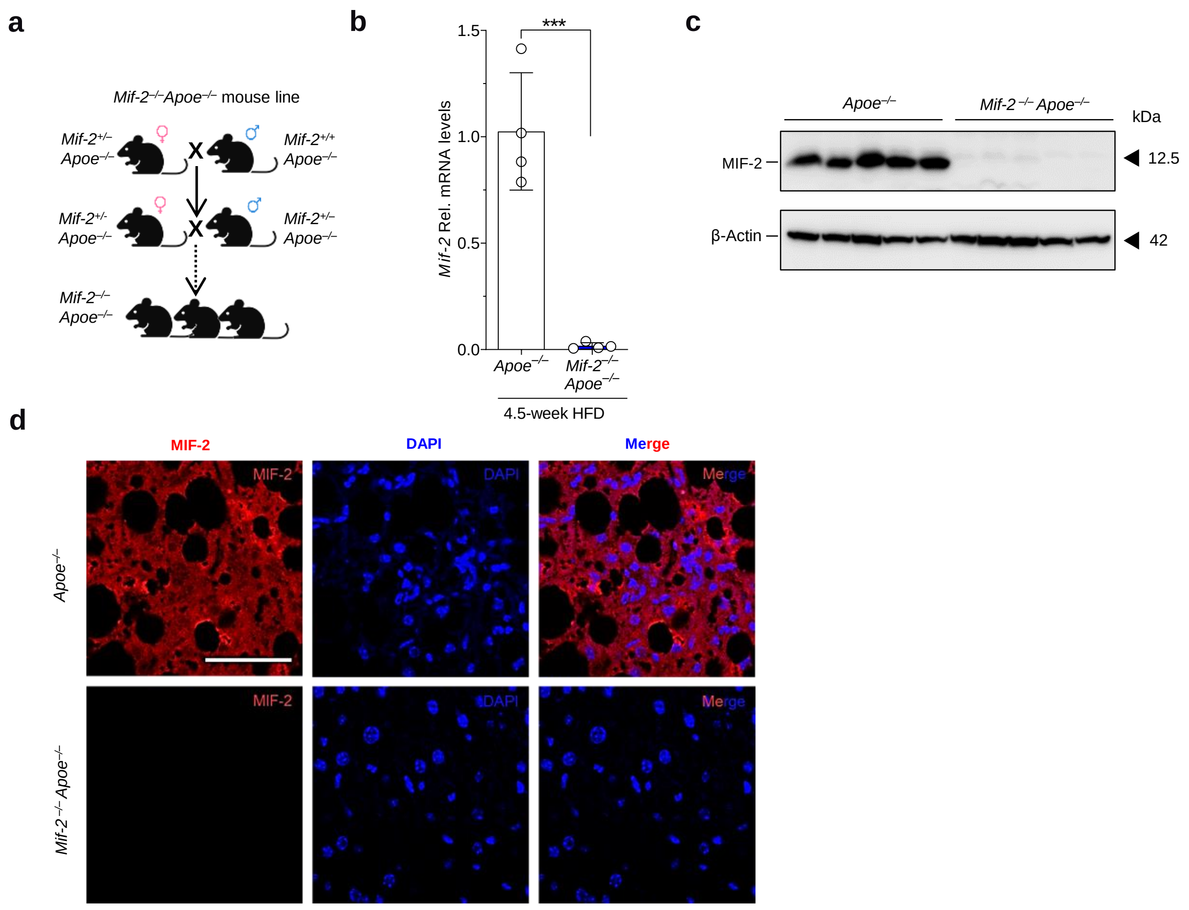

Supplementary Fig. 1: Generation and verification of the genetic *Mif-2* deletion in hyperlipidemic *Apoe*$^{-/-}$ mice. a Generation of *Mif-2*$^{-/-}$*Apoe*$^{-/-}$ mice. *Mif-2*$^{+/-}$*Apoe*$^{-/-}$ progeny were crossed with *Apoe*$^{-/-}$ mice and the resulting heterozygous mice were intercrossed to obtain homozygous mice. **b-d** Validation of the global deletion of *Mif-2* in heart tissues isolated from female *Apoe*$^{-/-}$ or from *Mif-2*$^{-/-}$ *Apoe*$^{-/-}$ mice fed HFD for 12 weeks as evaluated by RT-qPCR (**b**), by Western blotting using anti-MIF-2 antibody (**c**), or by immunofluorescence staining of paraffin liver sections using anti-MIF-2 and anti-Cy3 antibodies (**d**). n = 4 (**b**), n = 5 (**c**) mice per group. Scale bar: 50 µm (**d**). (**a**) was created in BioRender. Bernhagen, L. (2025) https://BioRender.com/c59v181. Values are means ± SD and were analyzed by unpaired two-tailed Student's t-test.

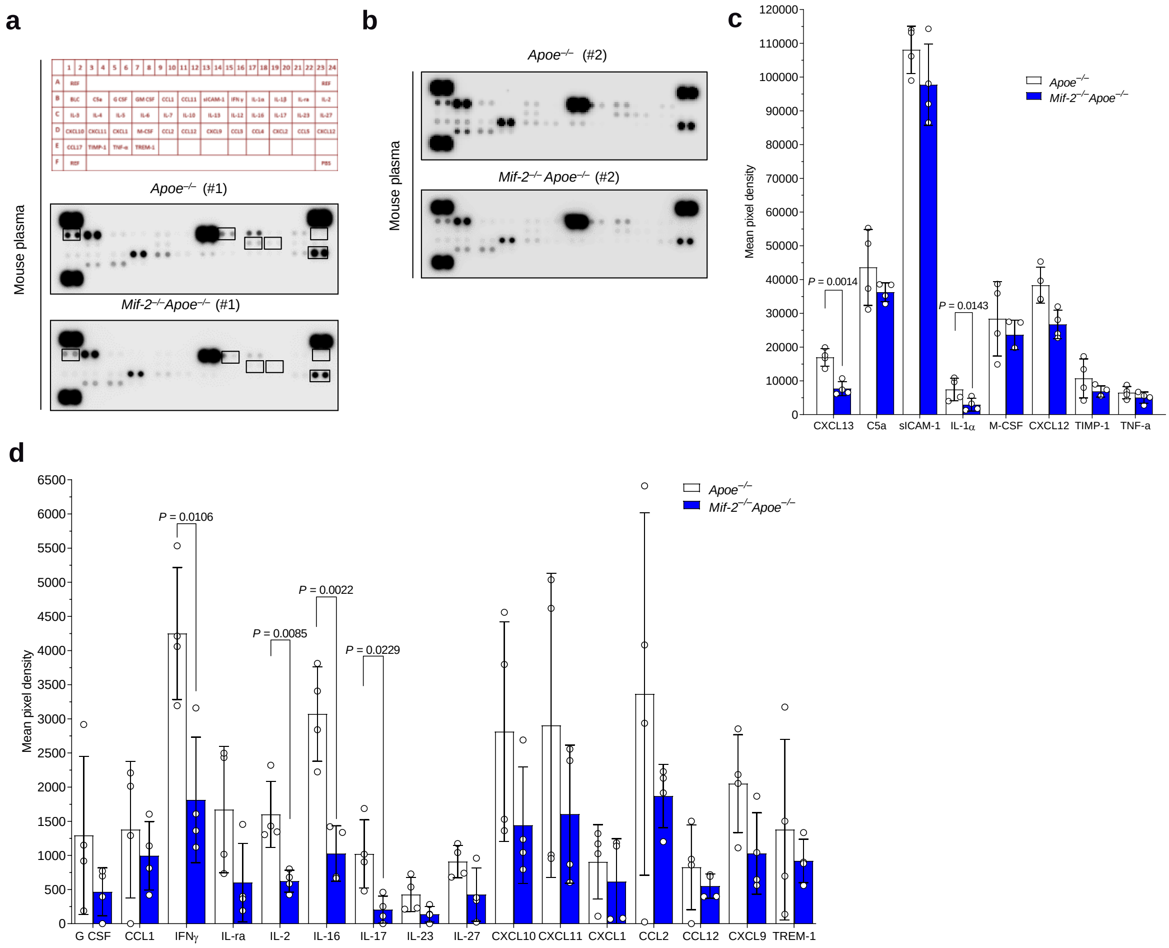

Supplementary Fig. 2: *Mif-2* deficiency leads to downregulated inflammatory cytokine levels in female *Apoe*$^{-/-}$ mice. a-b Two representative dot blots of plasma cytokines and chemokines from female *Apoe*$^{-/-}$ and *Mif-2*$^{-/-}$*Apoe*$^{-/-}$ mice fed a high fat, high-cholesterol diet (HFD) (Western diet) for 4.5 weeks. **c-d** Quantitative analysis of mouse cytokine array featuring 40 inflammatory/atherogenic cytokines/chemokines on plasma samples from both groups of mice and grouped in cytokines/chemokines with a relatively higher plasma abundance (**c**), and those with a relatively lower plasma abundance (**d**). Data (**c**, **d**) are shown as means ± SD from 4 mice per group performed in duplicate each and were analyzed with multiple unpaired t-test.

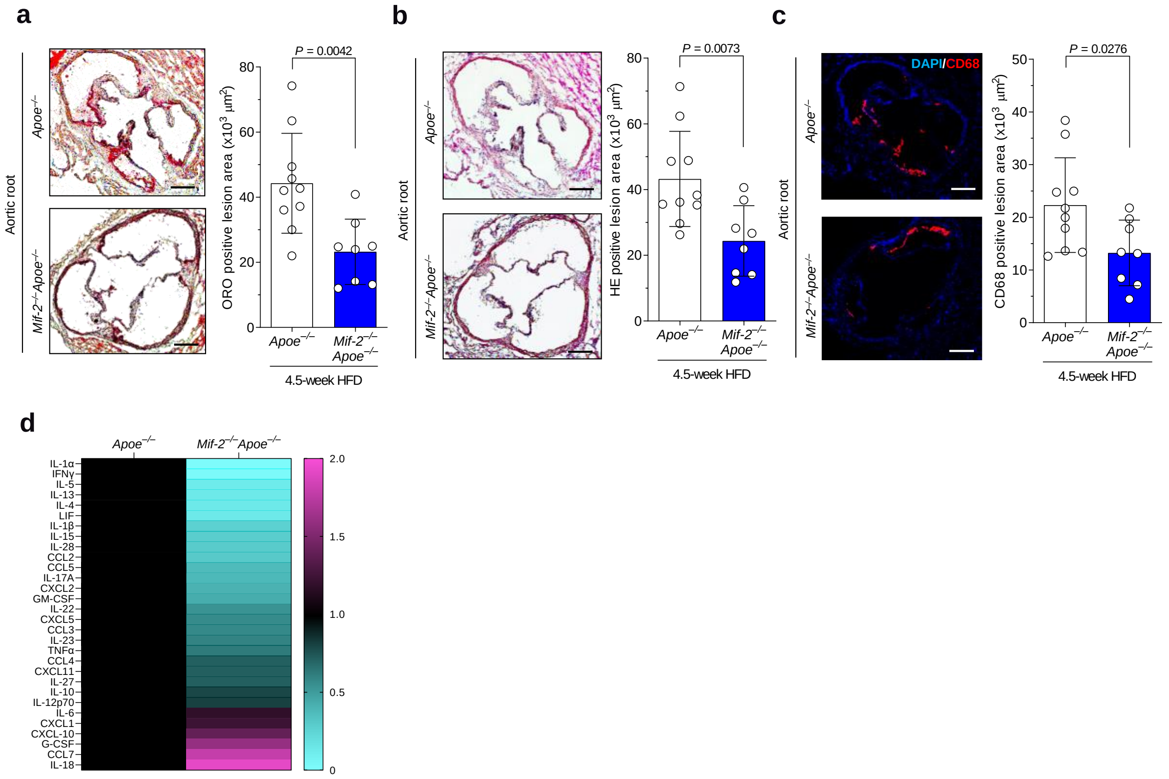

Supplementary Fig. 3: *Mif-2* deficiency attenuates early atherogenesis and vascular inflammation in male mice. a Representative Oil Red O (ORO) staining images of aortic roots from serial frozen sections (8 µm) of male *Apoe*$^{-/-}$ and *Mif-2*$^{-/-}$*Apoe*$^{-/-}$ mice fed a Western-type high fat high cholesterol diet (HFD) for 4.5 weeks and corresponding quantification results (12 sections per mouse). n = 8-10 mice per group. Scale bar: 250 µm. **b** Same as (**a**) except that hematoxylin-eosin (HE) staining was performed instead of ORO staining. **c** Representative images and quantification results of CD68^{+} macrophage content (red) in aortic root sections (8 µm) from male *Apoe*$^{-/-}$ and *Mif-2*$^{-/-}$ *Apoe*$^{-/-}$ mice fed HFD for 4.5 weeks (DAPI, blue). n = 8-10 mice per group. Scale bar: 250 µm. **d** Heatmap of multiplex cytokine array (36 ProcartaPlex mouse kit for cytokines, chemokines, and growth factors) in plasma of male *Apoe*$^{-/-}$ mice and *Mif-2*$^{-/-}$ *Apoe*$^{-/-}$ fed a HFD for 4.5 weeks performed with Luminex multiplex technology. Cytokines from *Apoe*$^{-/-}$ are normalized to 1 (first column, black) and were compared with those from *Mif-2*$^{-/-}$ *Apoe*$^{-/-}$ mice (second column). Up-regulated cytokines are shown in magenta, whereas down-regulated cytokines are shown in cyan. All values (**a**-**c**) are means ± SD and were analyzed by unpaired two-tailed Student's t-test.

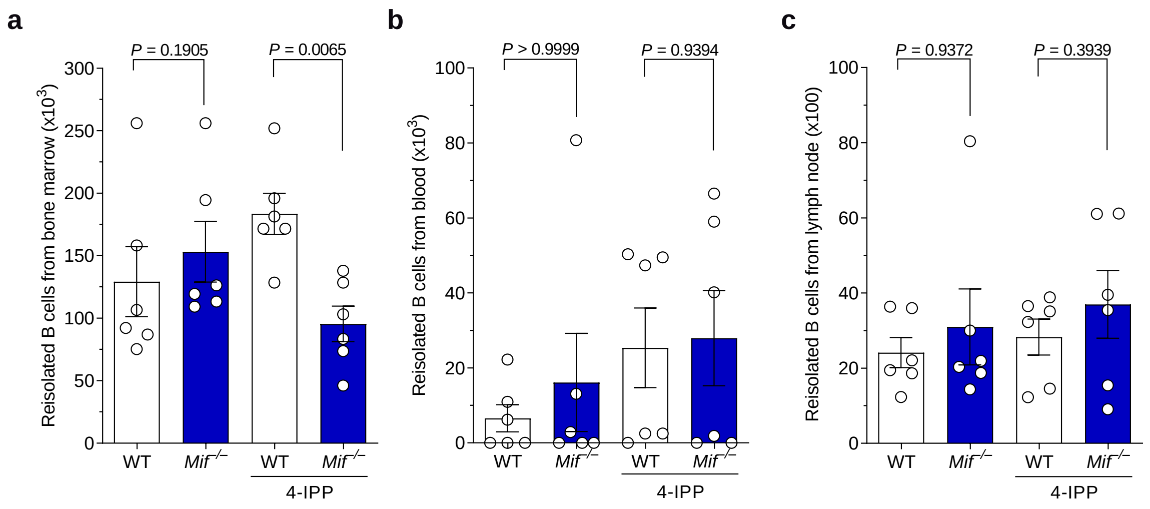

Supplementary Fig. 4: Quantification of B cells in bone marrow, blood, and lymph nodes after an *in vivo* B cell homing assay. Isolated primary B cells were stained with green cell tracker dye (CMFDA) and injected into the tail vein of *Mif* $^{-/-}$ or C57/BL6 control mice (WT) previously administered 4-IPP (2.5 mg/kg) or vehicle (saline containing 0.1% DMSO solvent) intraperitoneally. After 2 h, CMFD^{+} B cells in bone marrow (**a**), blood (**b**) and lymph nodes (**c**) were analyzed by flow cytometry. All values (**a**-**c**) are means ± SD and were analyzed by two-tailed Mann Whitney test.

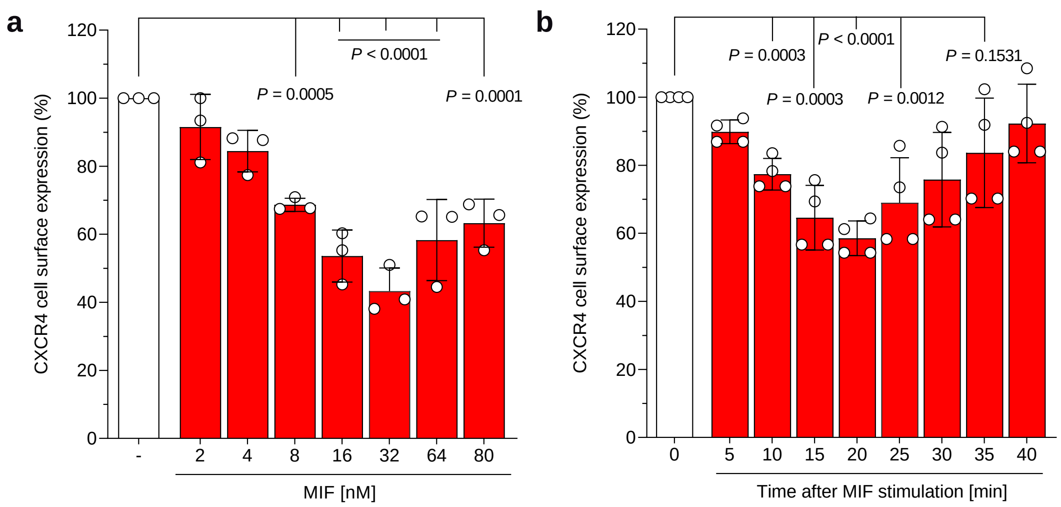

Supplementary Fig. 5: MIF induces the internalization of CXCR4 in a dose- and time-dependent manner. a MIF increases internalization of CXCR4 in primary B lymphocytes isolated from wildtype C57BL/6 mice in a dose-dependent manner. Isolated splenic B cells were stimulated with various concentrations of MIF for 20 min at 37°C, and cell surface expression of CXCR4 was analyzed by flow cytometry using APC-conjugated anti-CXCR4 antibody. **b** Time-course experiment of MIF-mediated CXCR4 internalization, in which B cells were stimulated with 32 nM of MIF. All values are shown as means ± SD; analysis was performed with one-way ANOVA with Dunnett's multiple comparisons test.

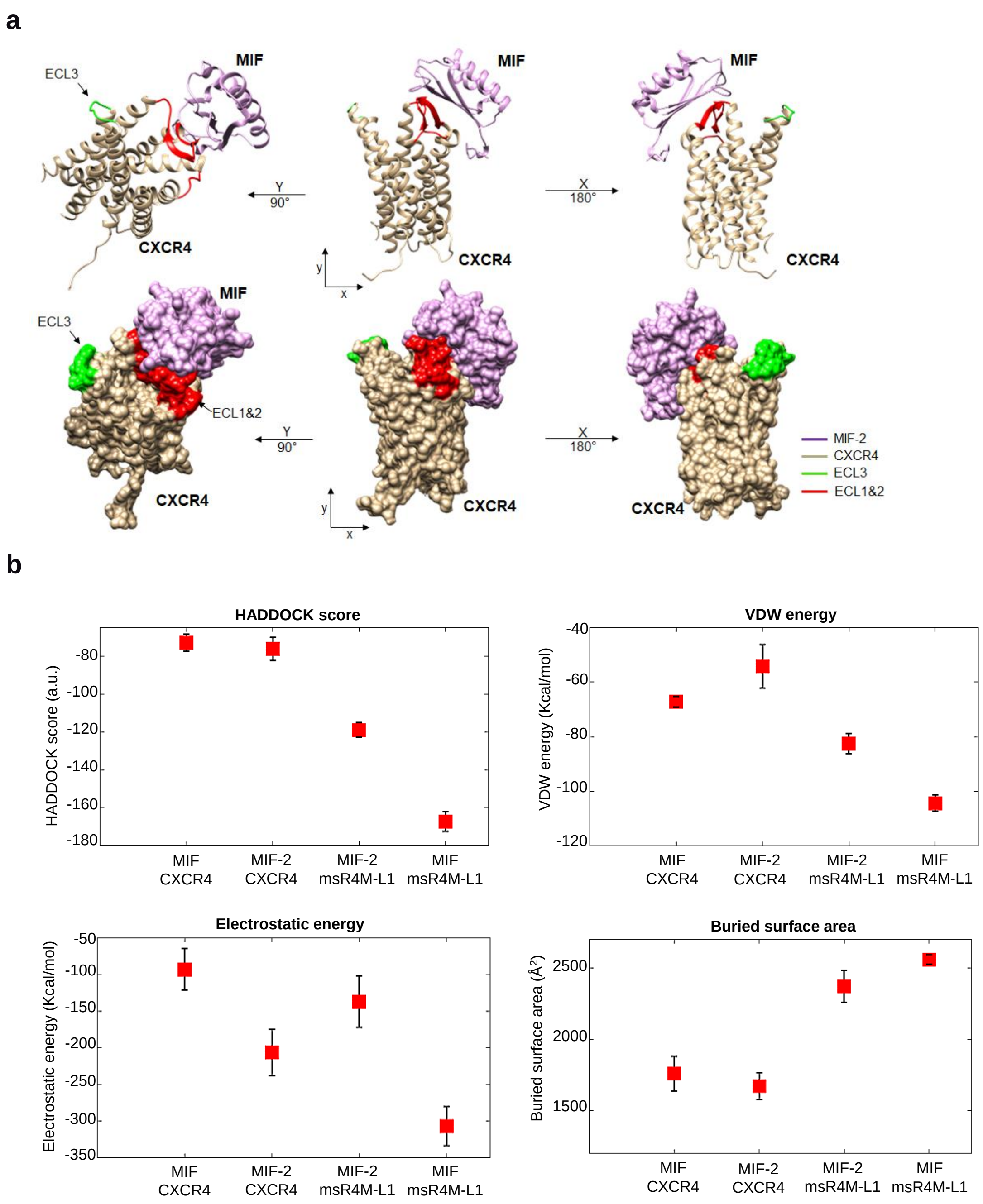

Supplementary Fig. 6: Statistical analysis of protein-protein docking simulation obtained by HADDOCK. a *In situ* molecular docking simulation between monomeric MIF (in purple) and CXCR4 (in yellow-gold (sandcastle)) using HADDOCK. The MIF/CXCR4 complex is shown in both ribbon (top) and surface area (bottom) views from different perspectives as indicated. The extracellular loops ECL1 and ECL2 in the CXCR4 sequence are shown in red, while ECL3 is indicated in green. **b** Comparison of the different HADDOCK scores of the selected complexes MIF/CXCR4, MIF-2/CXCR4, MIF-2/msR4M-L1, and MIF/msR4M-L1. The HADDOCK score corresponds to the weighted sum of intramolecular Van der Waals energy (VDW), electrostatic energy, and buried surface area. Average scores are calculated using the best 4 structures of each cluster.

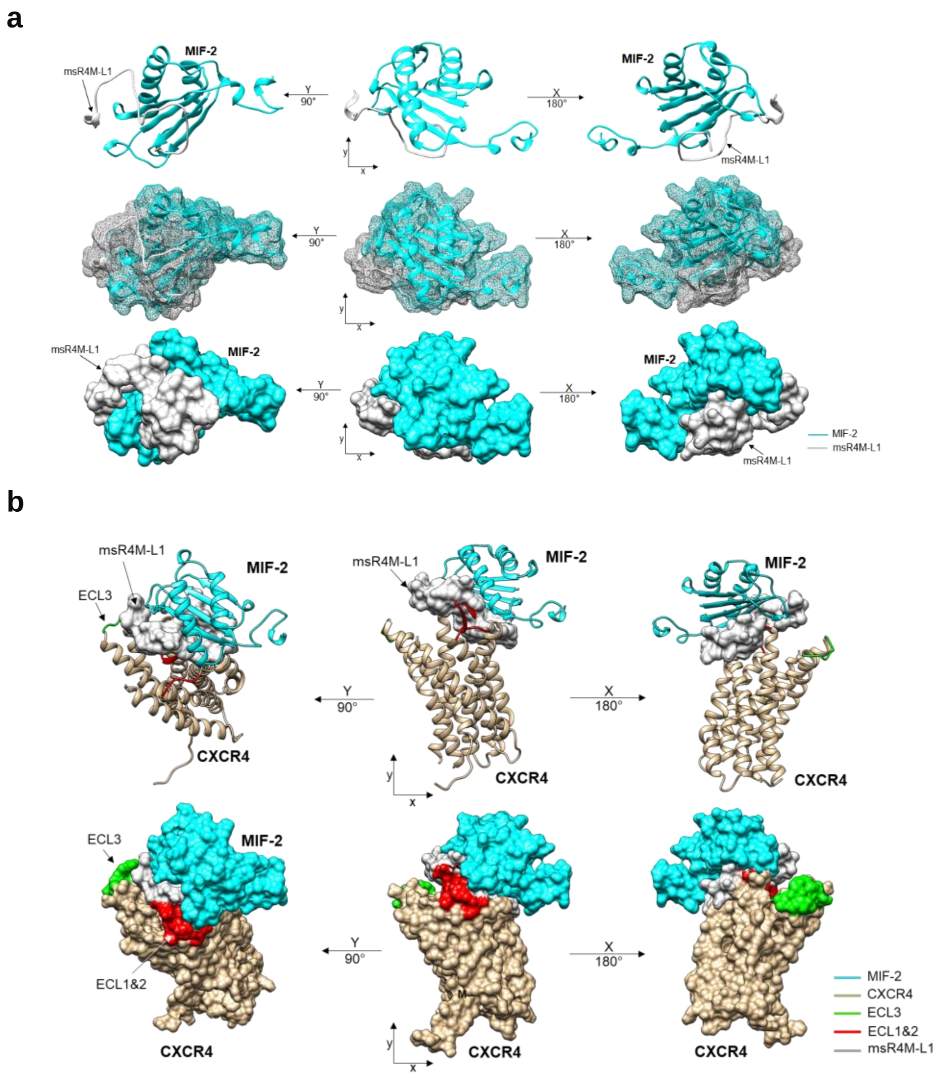

Supplementary Fig. 7: HADDOCK docking simulation between MIF-2 and the CXCR4 surrogate peptide msR4M-L1 and superimposition of MIF-2/CXCR4 and MIF-2/msR4M-L1 binding. a *In situ* molecular docking simulation between monomeric MIF-2 (in blue) and msR4M-L1(in grey) as determined by HADDOCK. The MIF-2/msR4M-L1 complex is shown in ribbon (top), ribbon/meshed (middle), and surface area (bottom) views from different perspectives as indicated. **b** Superposition of MIF-2 (blue), msR4M-L1 (grey) and CXCR4 (yellow gold (sandcastle)) shown in both ribbon (top), and surface area (bottom) views from different perspectives. The extracellular loops ECL1 and ECL2 of the CXCR4 sequence are shown in red, while ECL3 is indicated in green.

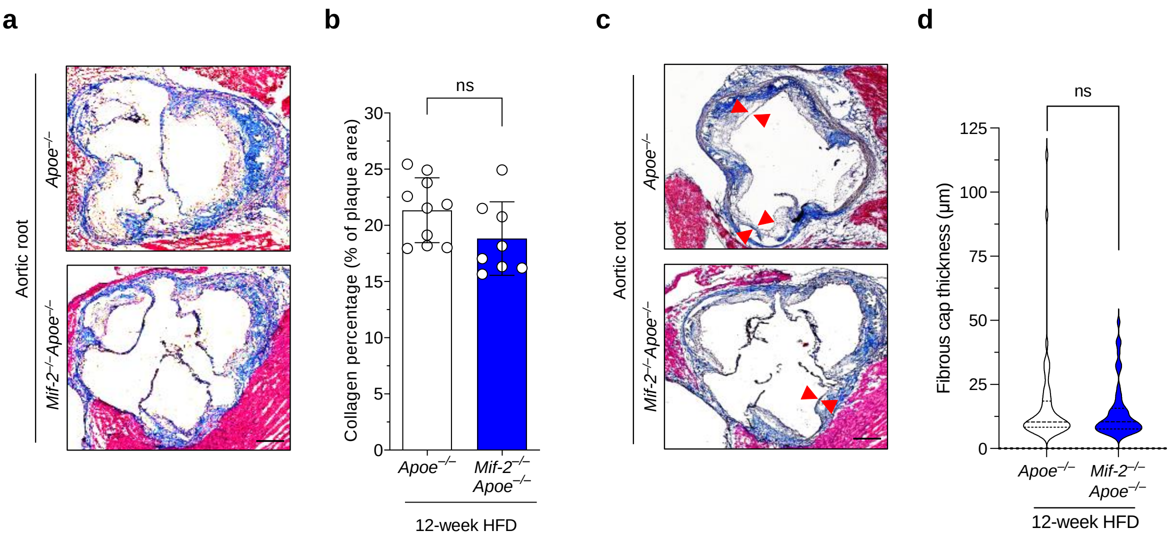

Supplementary Fig. 8: *Mif-2* deficiency does not affect the collagen content and fibrous cap thickness in hyperlipidemic *Apoe*$^{-/-}$ mice. a Representative images and (**b**) quantification results of Masson-stained frozen sections of aortic root from *Apoe*$^{-/-}$ and *Mif-2*$^{-/-}$*Apoe*$^{-/-}$ mice after 12-week Western-type high fat high cholesterol diet (HFD). (*Apoe*$^{-/-}$: n=10, *Mif-2*$^{-/-}$*Apoe*$^{-/-}$: n=8, scale bar: 200 μm). **c-d** Same as (**a**, **b**) except that representative images (**c**) and the quantification (**d**) are for the relative fibrous cap thickness. Scale bar: 200 μm. Fibrous caps are indicated with red arrows, which are next to the necrotic core regions within the plaque (blue staining). (*Apoe*$^{-/-}$: n=39, *Mif-2*$^{-/-}$*Apoe*$^{-/-}$: n=37). The thickness is indicated in μm. Values are means ± SD and were analyzed by two-tailed Student's t-test (**b**) and Kolmogorov-Smirnov test (**d**); ns, not significant.

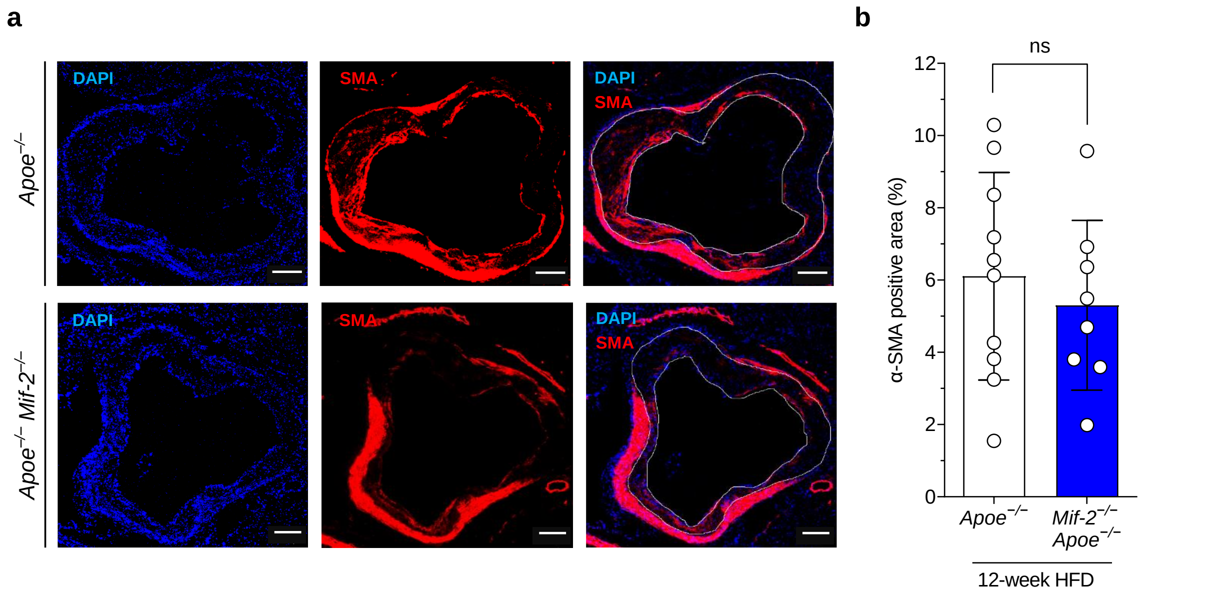

Supplementary Fig. 9: *Mif-2* deficiency does not alter the smooth muscle cell content in aortic lesions of hyperlipidemic *Apoe*$^{-/-}$ mice. a Representative images and (**b**) quantification results of α-SMA+ cells (red) of aortic root from *Apoe*$^{-/-}$ and *Mif-2*$^{-/-}$*Apoe*$^{-/-}$ mice after 12-week Western-type high fat-high diet (HFD) (DAPI, blue). (*Apoe*$^{-/-}$: n=10, *Mif-2*$^{-/-}$*Apoe*$^{-/-}$: n=8). Scale bar: 200 µm. α-SMA+ area was quantified in the intimal / lesional area (see circled areas in merged images). Values are means ± SD and were analyzed by unpaired two-tailed Student's t-test; ns, not significant.

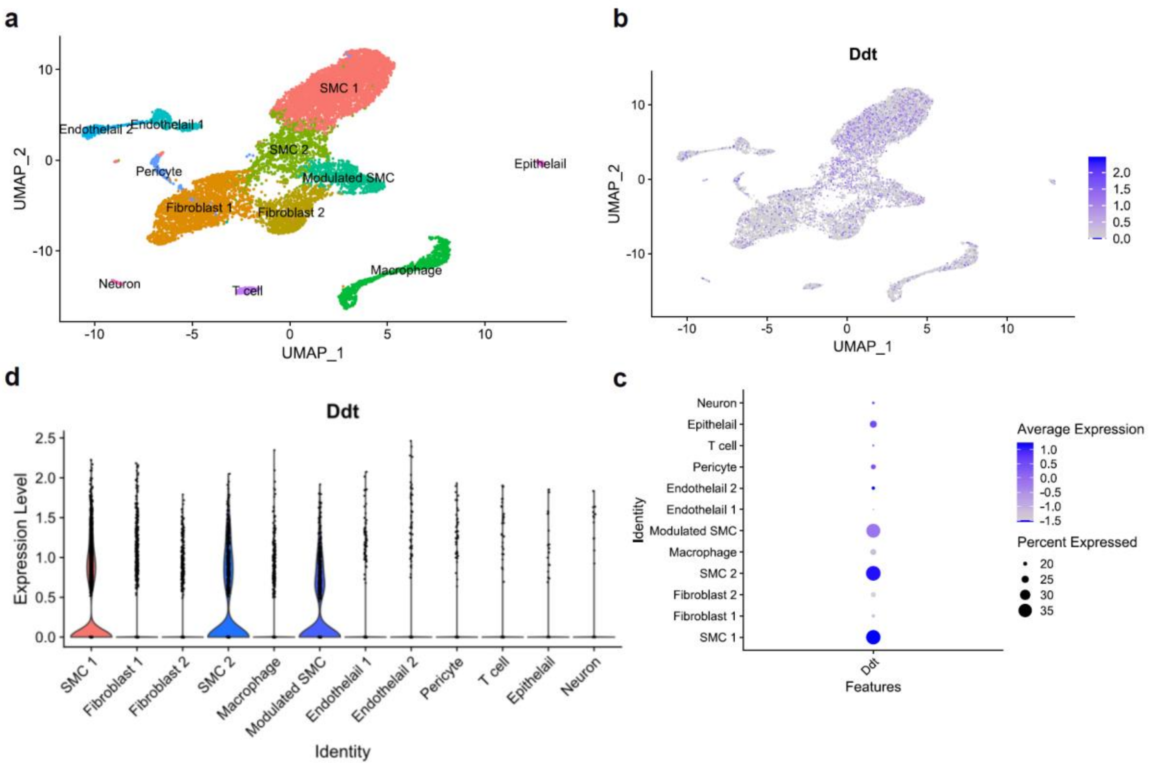

Supplementary Fig. 10: Re-analysis of single-cell RNAseq data from aortic root of *Apoe*$^{-/-}$ mice subjected to 8 and 16 weeks of HFD reveals a prominent expression pattern of Mif-2 in smooth muscle cells. The single-cell RNA-seq dataset GSE131780 was downloaded from GEO (Gene Expression Omnibus), and *Mif-2* (designated as *Ddt* in the data set) expression was re-analyzed in both SMCs and non-SMCs of *Apoe*$^{-/-}$ mice after 8 or 16 weeks of HFD (Wirka et al, *Nat Med* 2019[1]). **a** Uniform manifold approximation and projection (UMAP) visualization of single cell clusters from aortas of *Apoe*$^{-/-}$ mice after 8 or 16 weeks of HFD. Different cell clusters are represented by different colors as indicated. **b** Feature plot showing the expression of *Mif-2* (designated as *Ddt* in the data set) in different single cell clusters. **c** Violin plot showing the expression of *Mif-2* (*Ddt*) in different atherogenic cell populations. **d** Same as (**c**) except that *Mif-2* (*Ddt*) expression is depicted as dot plot. Dot size represents the percentage of cells expressing *Ddt*, and color intensity indicates the average expression level. SMCs, vascular smooth muscle cells.

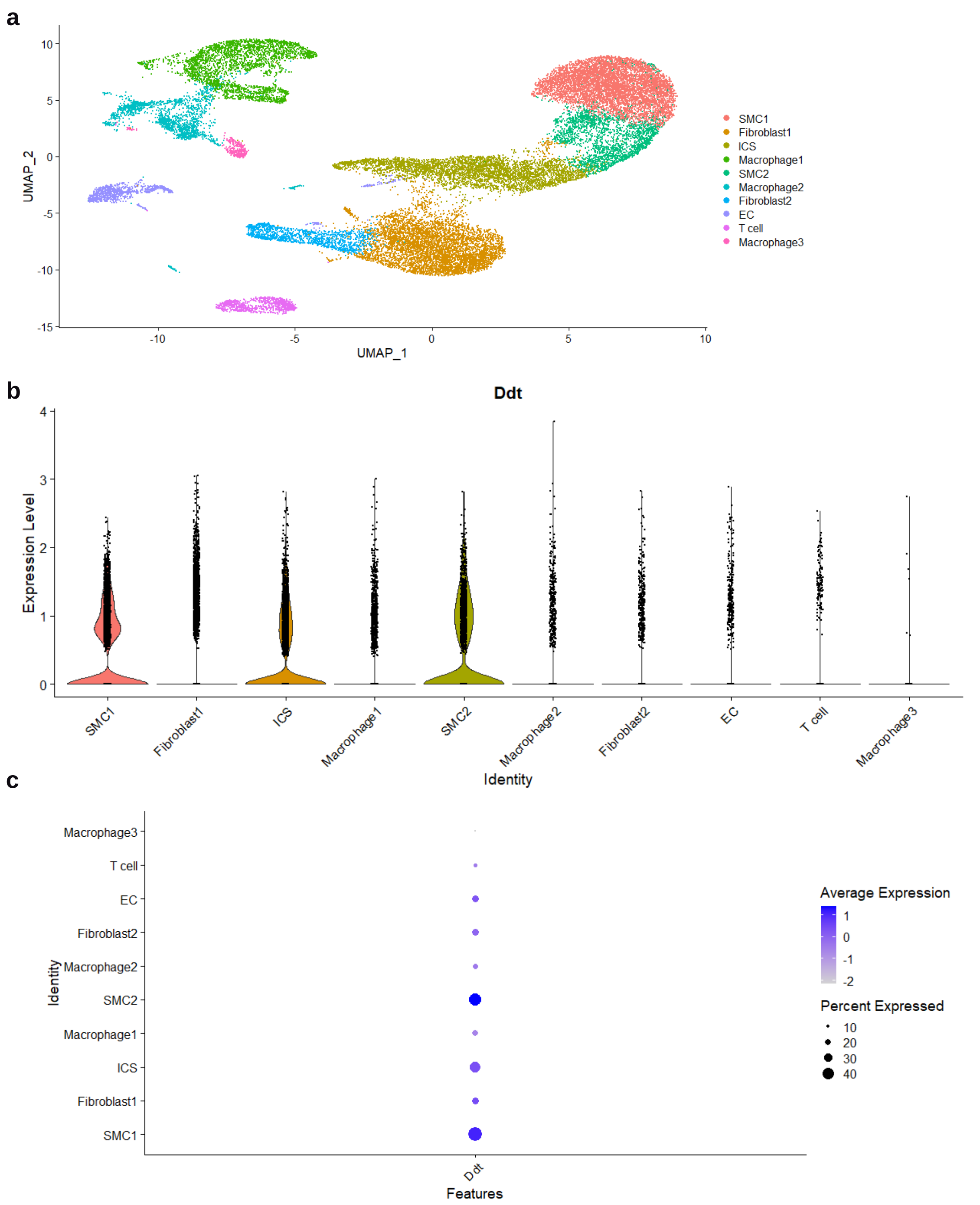

Supplementary Fig. 11: Re-analysis of single-cell RNAseq data from aortic root of *Ldlr*$^{-/-}$ mice subjected to HFD reveals a prominent expression pattern of Mif-2 in smooth muscle cells. The single-cell RNA-seq dataset GSE155514 was downloaded from GEO (Gene Expression Omnibus), and *Mif-2* (designated as *Ddt*) expression was re-analyzed in both SMCs and non-SMCs of *Ldlr* $^{-/-}$ mice after 8, 16, and 26 weeks of HFD (Pan et al, *Circulation* 2020[2]). **a** Uniform manifold approximation and projection (UMAP) visualization of single cell clusters from aortas of *Ldlr* $^{-/-}$ mice after 8, 16 and 26 weeks of HFD. Different cell clusters are represented by different colors as indicated. **b** Violin plot showing the expression of *Mif-2* (*Ddt*) in different atherogenic cell populations, with most abundant expression seen in SMC-type cells (SMC1, ICS, SMC2). **c** Same as (**b**) except that *Mif-2* (*Ddt*) expression is depicted as dot plot. Dot size represents the percentage of cells expressing *Ddt*, and color intensity indicates the average expression level. SMCs, vascular smooth muscle cells; ICS, SMC-derived intermediate cell state, also called SEM (stem cell, endothelial cell, monocyte).

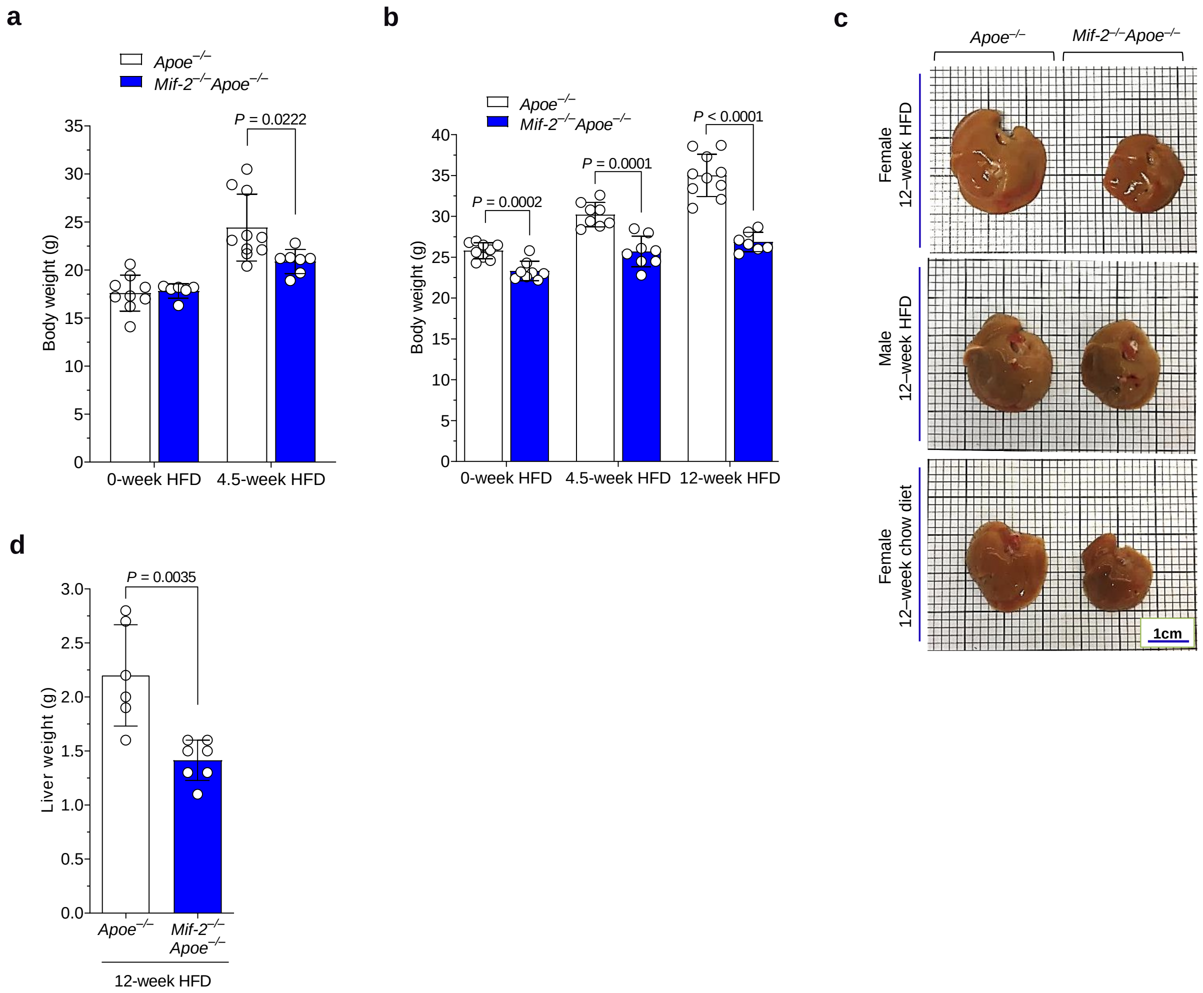

Supplementary Fig. 12: *Mif-2* knockout leads to weight loss and smaller liver size in male and female *Apoe*$^{-/-}$ mice. a *Mif-2*$^{-/-}$*Apoe*$^{-/-}$ mice had lower body weight than *Apoe*$^{-/-}$ mice after 4.5 weeks of HFD (*Apoe*$^{-/-}$: n=9, *Mif-2*$^{-/-}$*Apoe*$^{-/-}$: n=6 (0-week HFD); *Apoe*$^{-/-}$: n=6, *Mif-2*$^{-/-}$*Apoe*$^{-/-}$: n=7 (4.5-week HFD)). **b** Male *Apoe*$^{-/-}$ and *Mif-2*$^{-/-}$*Apoe*$^{-/-}$ mice were fed a Western-type high cholesterol, high fat diet (HFD) for 4.5 and 12 weeks. Significant differences in body weight were observed between both groups of male mice after 4.5- and 12-week HFD and before the onset of the HFD (*Apoe*$^{-/-}$: n=9, *Mif-2*$^{-/-}$*Apoe*$^{-/-}$: n=8 (0-week HFD); *Apoe*$^{-/-}$: n=8, *Mif-2*$^{-/-}$*Apoe*$^{-/-}$: n=8 (4.5-week HFD); *Apoe*$^{-/-}$: n=10, *Mif-2*$^{-/-}$*Apoe*$^{-/-}$: n=7 (12-week HFD)). **c** Representative images of the livers of female and male *Apoe*$^{-/-}$ and *Mif-2*$^{-/-}$*Apoe*$^{-/-}$ mice after both 12-week HFD and 12-week chow diet. Livers of *Mif-2*$^{-/-}$*Apoe*$^{-/-}$ mice appeared generally smaller in both sexes. Scale bar: 1 cm. **d** Quantification results showing that liver weight of male *Mif-2*$^{-/-}$*Apoe*$^{-/-}$ mice was significantly decreased after 12-week HFD compared with *Apoe*$^{-/-}$ mice (*Apoe*$^{-/-}$: n=6, *Mif-2*$^{-/-}$*Apoe*$^{-/-}$: n=7). Values are means ± SD and were analyzed by unpaired two-tailed Student's t-test (**a**, **b**) and two-tailed Mann Whitney test (**d**).

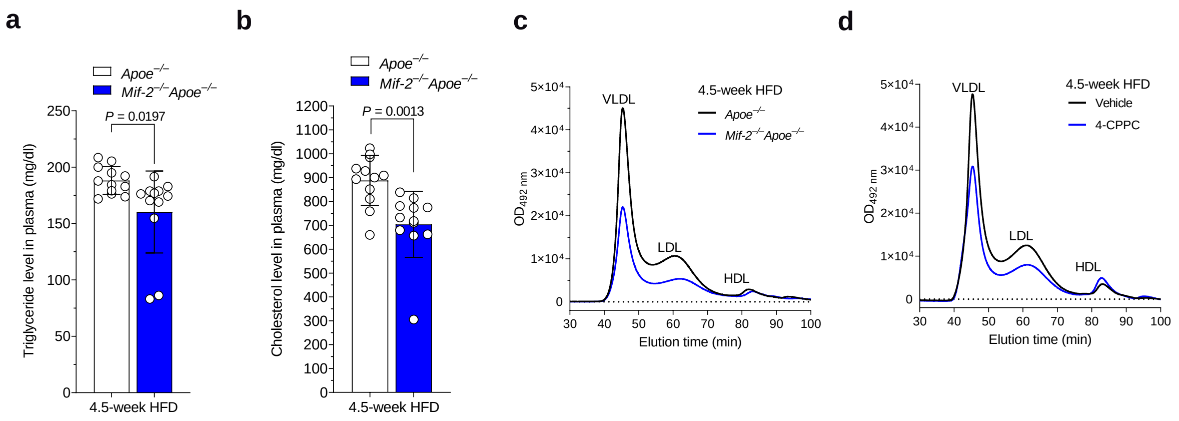

Supplementary Fig. 13: *Mif-2*$^{-/-}$*Apoe*$^{-/-}$ mice have lower circulating plasma lipid levels. Reduced Levels of plasma triglycerides (**a**) and plasma total cholesterol (**b**) from *Apoe*$^{-/-}$ and *Mif-2* $^{-/-}$*Apoe*$^{-/-}$ mice fed a HFD for 4.5 weeks. n = 12 mice per group. **c-d** *Mif-2* deficiency or pharmacological blockade reduces atherogenic lipoproteins in plasma after 4.5-week HFD. (**a-c**) Female *Apoe*$^{-/-}$ and *Mif-2*$^{-/-}$*Apoe*$^{-/-}$ mice fed HFD for 4.5 weeks. (**d**) Male *Apoe*$^{-/-}$ mice treated with 4-CPPC *versus* vehicle after 4.5-week HFD. Values are means ± SD and were analyzed by unpaired two-tailed Student's t-test (**a**, **b**).

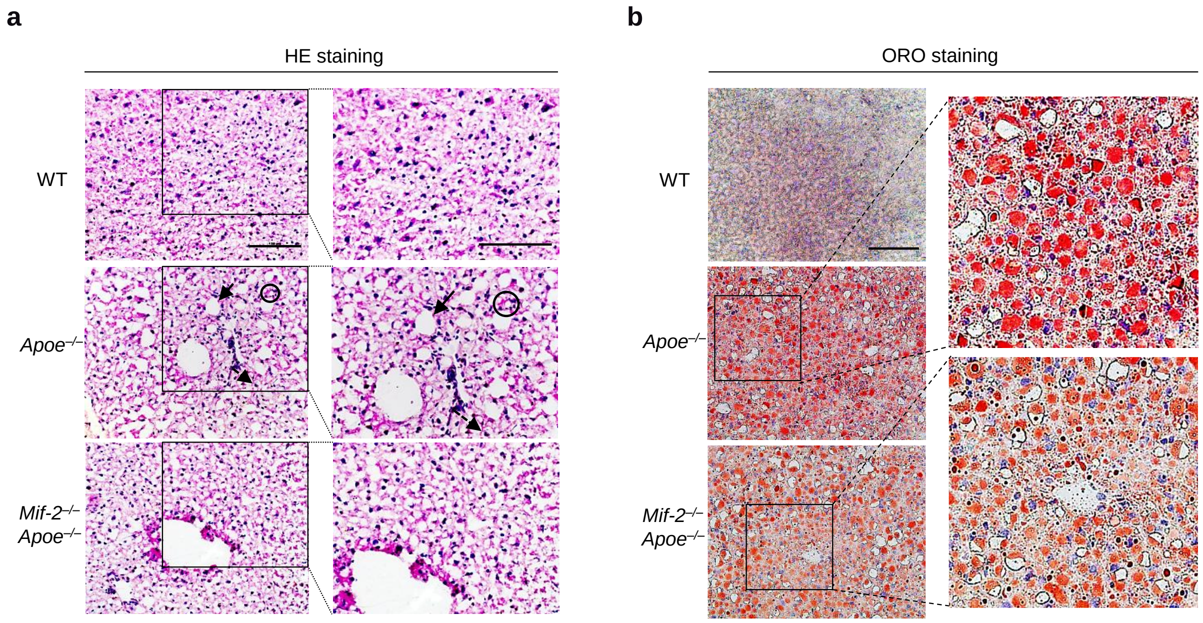

Supplementary Fig. 14: *Mif-2* knockout alleviates hepatic lipid accumulation in hyperlipidemic *Apoe*$^{-/-}$ mice. a Determination of hepatic lipid accumulation by HE staining using frozen sections. Representative liver images of *Mif-2*$^{-/-}$*Apoe*$^{-/-}$ mice show lower lipid accumulation compared with *Apoe*$^{-/-}$ mice. Classic structures, for example macrovesicular steatosis (bold arrows), microvesicular steatosis (dotted line arrows), and clusters (aggregates) of inflammatory cells (within circles) could be identified. Right-hand panel, magnified images. **b** Analysis of hepatic lipid accumulation by ORO staining using frozen sections. Similar to HE staining, ORO-stained liver images from *Mif-2*$^{-/-}$*Apoe*$^{-/-}$ mice showed less accumulation of neutral lipids compared with *Apoe*$^{-/-}$ mice. Enlarged insets on the right panel. The figure shows magnified views of the analysis shown in **Fig. 5c**. Control images from wildtype (WT) mice (top left) without atherogenic background and Western-type high fat diet (HFD) demonstrate the effects of the atherogenic phenotype on hepatosteatosis. Scale bar: 200 µm.

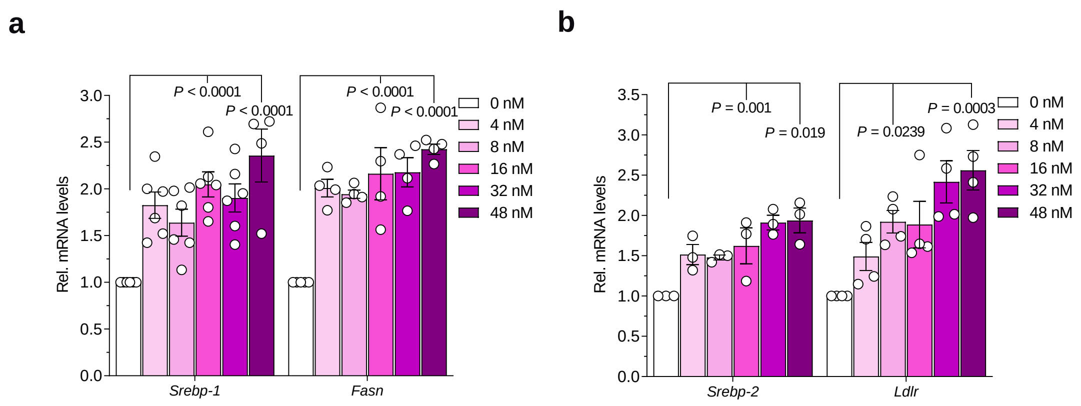

Supplementary Fig. 15: MIF-2 stimulation increases the gene expression of *SREBPs* and their lipogenic downstream target transcripts in Huh-7 cells. mRNA levels of *Srebp-1/Fasn* (**a**) and *Srebp-2*/*Ldlr* (**b**) were measured by RT-qPCR following stimulation of Huh-7 cells with various concentrations of MIF-2 for 24 h (*Srebp1*: n=5; *Fasn*: n=4). (*Srebp2*: n=3; *Ldlr*: n=4). All values are shown as means ± SD and analysis was performed with one-way ANOVA with Dunnett's multiple comparisons test.

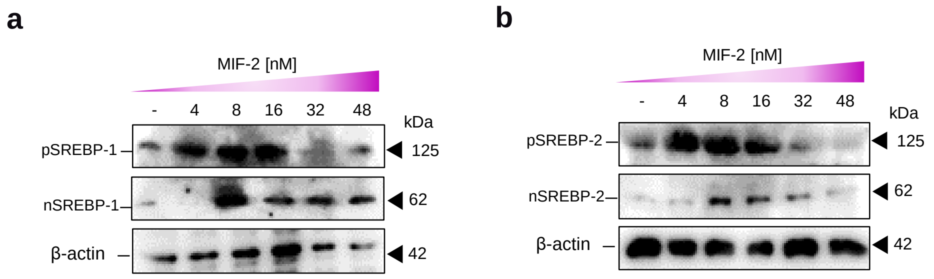

Supplementary Fig. 16: MIF-2 promotes proteolytic cleavage and subsequent activation of SREBP-1/2 in primary hepatocytes. Isolated primary hepatocytes from WT mice were stimulated with different concentrations of rMIF-2 for 24 h, and protein levels of pSREBP-1 and nSREBP-1 (**a**) as well as pSREBP-2 and nSREBP-2 (**b**) were analyzed by Western blot using anti-SREBP-1 and anti-SREBP-2 antibodies. β-actin was run as a loading standard.

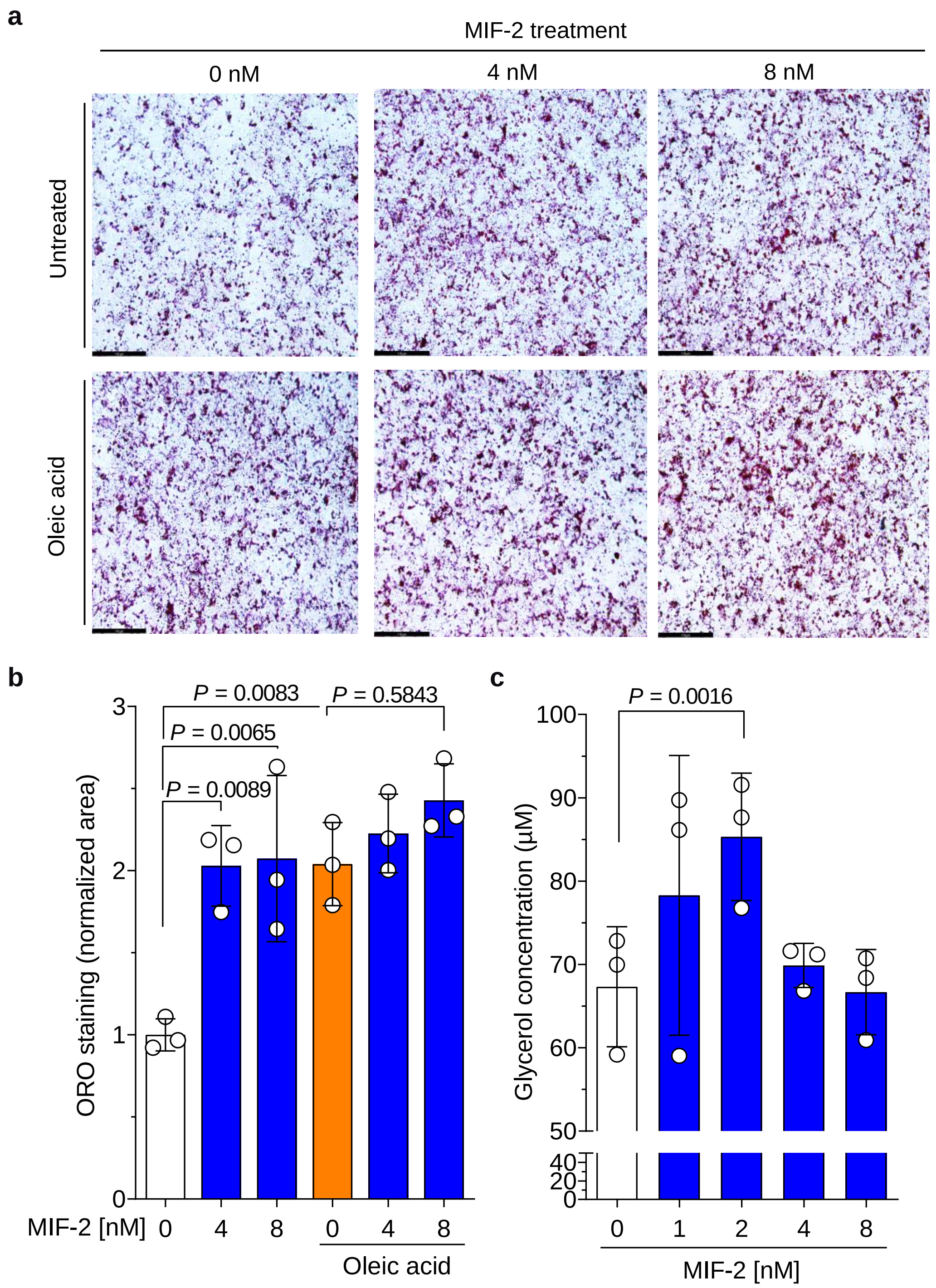

Supplementary Fig. 17: *In vitro* steatosis and triglyceride assays indicate that MIF-2 modulates lipid accumulation and triglyceride synthesis in Huh-7 hepatocytes. **a** Representative images of oil red O (ORO) staining show lipid accumulation in Huh-7 cells treated with varying concentrations of MIF-2 (0, 4, and 8 nM), either in the absence (top row) or presence (bottom row) of 1 mM oleic acid, as assessed by the *in vitro* steatosis assay. Red/violet staining indicates areas of lipid accumulation. Scale bars: 120 µm. **b** Quantification of ORO staining (normalized area) according to panel (**a**). **c** Determination of total glycerol concentrations in Huh-7 cells as a readout for cellular triglycerides following MIF-2 treatment at different concentrations (0, 1, 2, 4, and 8 nM) using *in vitro* 'triglyceride assay'. Data are expressed as mean ± SD (n = 3). Statistical analysis was determined using one-way ANOVA with Tukey's multiple comparisons (**b**) or with Holm-Šídák's multiple comparisons test (**c**).

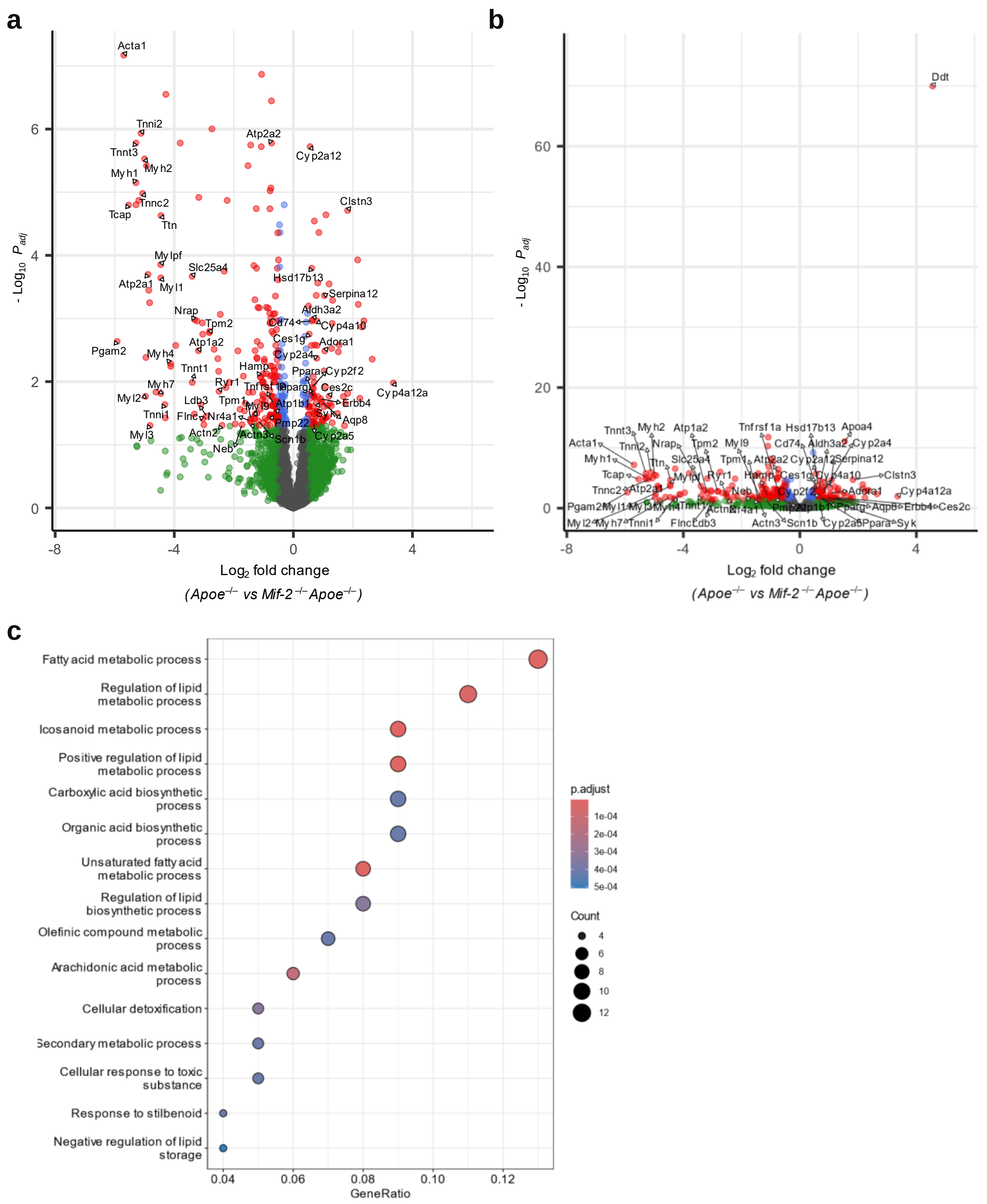

Supplementary Fig. 18: Bulk RNA sequencing of liver tissue from *Apoe*$^{-/-}$ and *Mif-2*$^{-/-}$ *Apoe*$^{-/-}$ mice. Total RNA was isolated from the livers of *Apoe*$^{-/-}$ and *Mif-2*$^{-/-}$*Apoe*$^{-/-}$ mice (n=7 per group) after 12 weeks of HFD and used for liver transcriptome analysis by bulk RNAseq. **a** Volcano plot illustrating differential gene expression (DEG). Right side: transcripts up-regulated in *Apoe*$^{-/-}$, down-regulated in *Mif-2*$^{-/-}$*Apoe*$^{-/-}$ mice; left side: transcripts down-regulated in *Apoe*$^{-/-}$, up-regulated in *Mif-2*$^{-/-}$*Apoe*$^{-/-}$ mice. Red dots represent genes meeting significance criteria (P_{adj}<0.05; Log_2-fold change>0.5). Non-significant genes with log_2-fold change<0.5 are depicted as dark-gray dots. Green dots represent genes with log_2-fold change>0.5 and P_{adj}>0.05, blue dots are log_2-fold change<0.5 and P_{adj}<0.05. **b** Same as (A), except that the y-axis of the Volcano plot is differently scaled to include the *Mif-2* gene (here annotated at *Ddt*). **c** Dot plot representation of a GO pathway analysis showing terms significantly enriched in *Mif-2*$^{-/-}$*Apoe*$^{-/-}$ compared to *Apoe*$^{-/-}$ mice. The size of dots (counts) represents the number of genes populating a term; color code indicates significance level P_{adj}. Pathways were sorted according to gene ratio (GeneRatio).

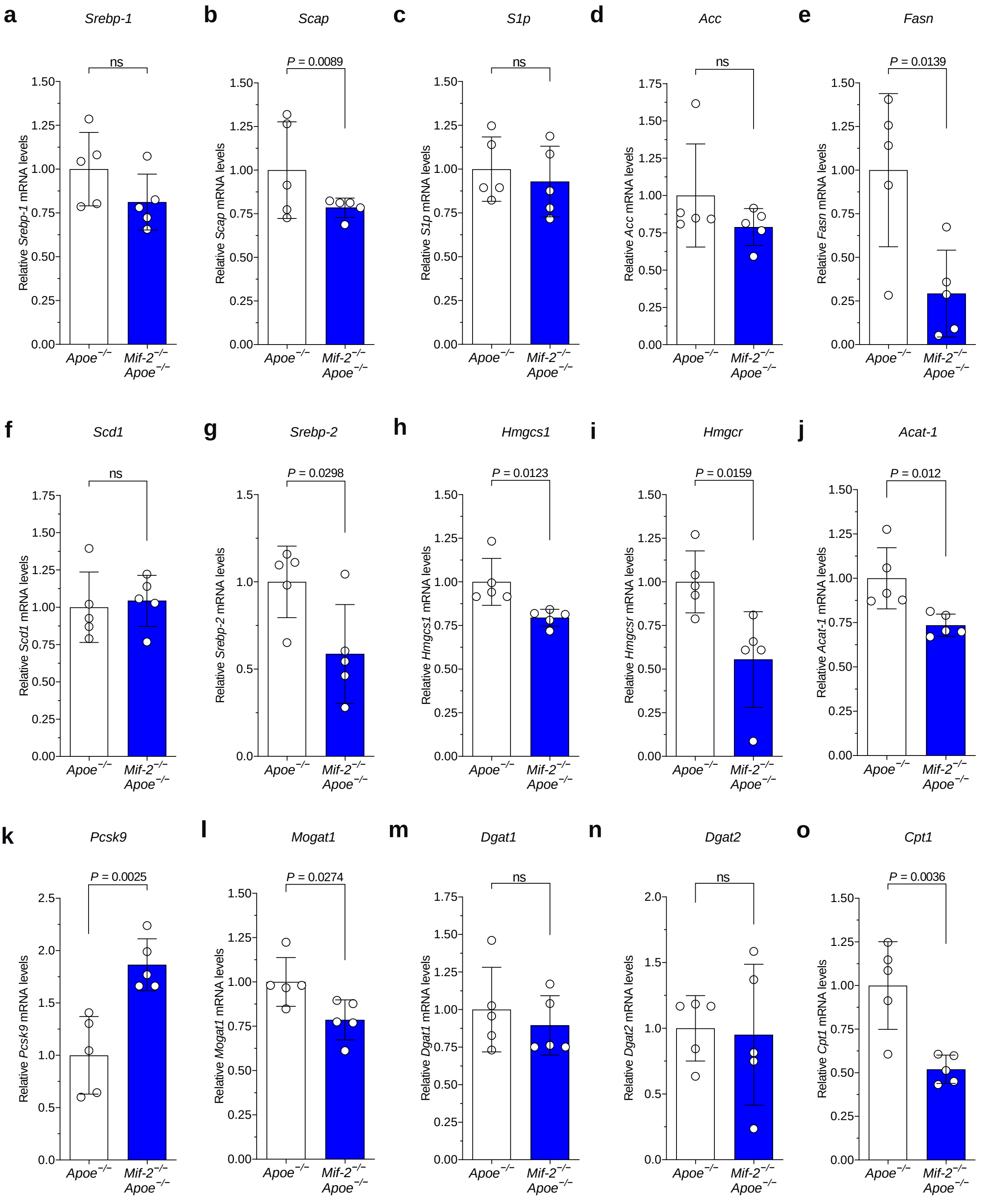
a
Srebp-1
ns
Relative Srebp-1 mRNA levels
b
Scap
P = 0.0089
Relative Scap mRNA levels
c
S1p
ns
Relative S1p mRNA levels
d
Acc
ns
Relative Acc mRNA levels
e
Fasn
P = 0.0139
Relative Fasn mRNA levels
f
Scd1
ns
Relative Scd1 mRNA levels
g
Srebp-2
P = 0.0298
Relative Srebp-2 mRNA levels
h
Hmgcs1
P = 0.0123
Relative Hmgcs1 mRNA levels
i
Hmgcr
P = 0.0159
Relative Hmgcsr mRNA levels
j
Acat-1
P = 0.012
Relative Acat-1 mRNA levels
k
Pcsk9
P = 0.0025
Relative Pcsk9 mRNA levels
l
Mogat1
P = 0.0274
Relative Mogat1 mRNA levels
m
Dgat1
ns
Relative Dgat1 mRNA levels
n
Dgat2
ns
Relative Dgat2 mRNA levels
o
Cpt1
P = 0.0036
Relative Cpt1 mRNA levels
Apoe−/−
Mif-2−/− Apoe−/−

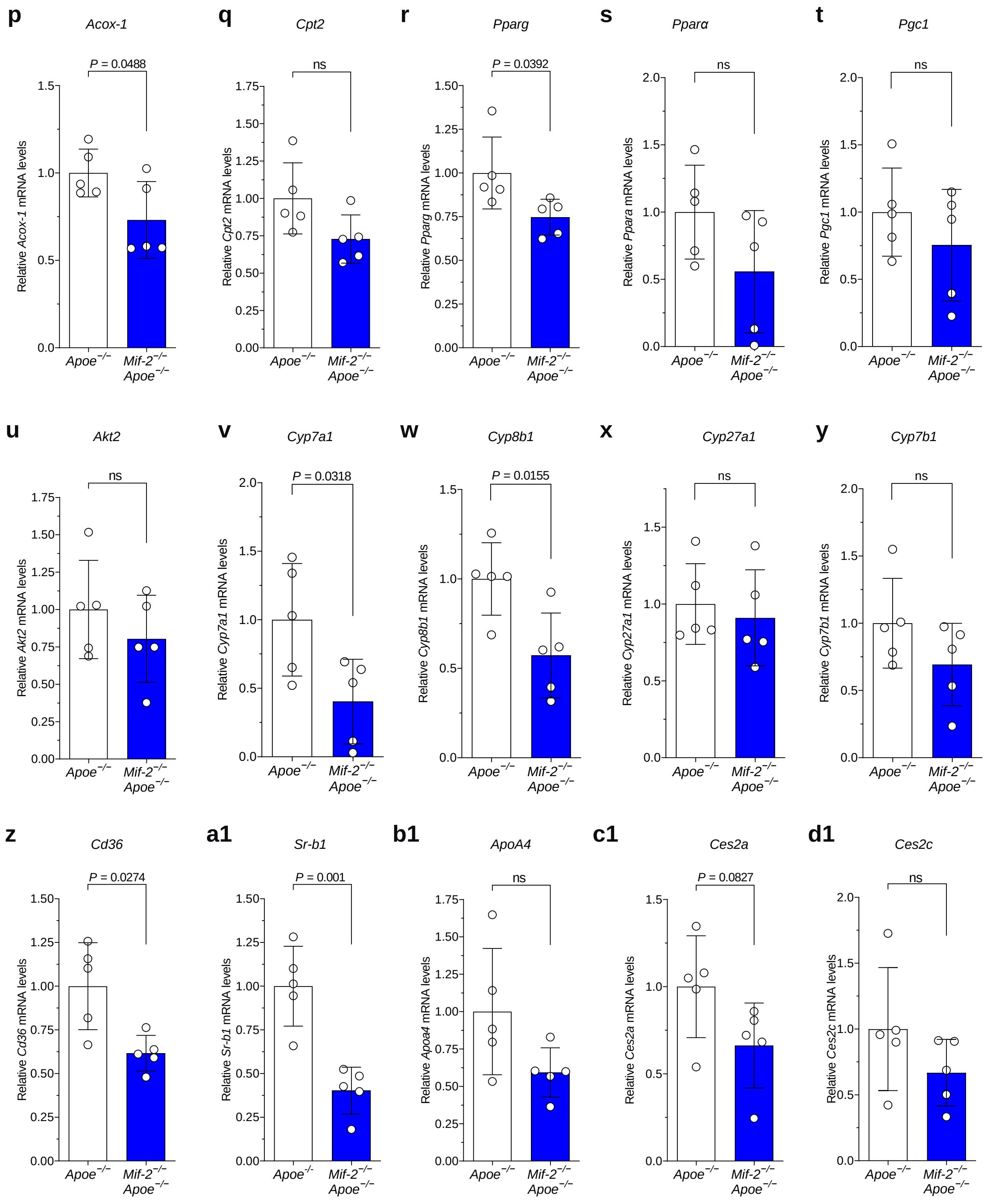

Supplementary Fig. 19: mRNA expression of lipogenic and fatty acid metabolism-related genes in liver tissue of *Apoe*$^{-/-}$ and *Mif-2*$^{-/-}$*Apoe*$^{-/-}$ mice as determined by RT-qPCR. Total RNA was extracted from the livers of *Apoe*$^{-/-}$ and *Mif-2*$^{-/-}$*Apoe*$^{-/-}$ mice (n = 5 per group) after 12 weeks of HFD. The mRNA levels of various genes associated with lipogenesis were quantified by RT-qPCR and are expressed as relative mRNA expression levels. All values are expressed as means ± SD and statistically analyzed using unpaired two-tailed t-test. The genes are grouped and classified based on their roles in lipid metabolism as follows: (**a-f**) lipogenesis and fatty acid synthesis; (**g-k**) cholesterol synthesis and regulation; (**l-n**) triglyceride synthesis; (**o-q**) fatty acid oxidation; (**r-u**) lipid metabolism regulation and signaling; (**v-y**) bile acid synthesis; (**z-d1**) lipid hydrolysis, absorption, and transport. Data are presented as means ± SD and were analyzed by two-tailed Student's t-test. Gene abbreviations: *Srebp1*,

sterol-regulatory element-binding protein 1; *Scap*, Srebp cleavage-activating protein; *S1p*, site-1 protease; *Acc*, acetyl-CoA carboxylase; *Fasn*, fatty acid synthase; *Scd1*, stearoyl-CoA desaturase 1; Srebp2, sterol-regulatory element-binding protein 2; *Hmgcs1*, 3-hydroxy-3-methylglutaryl-CoA synthase 1; *Hmgcr*, 3-hydroxy-3-methylglutaryl-CoA reductase; *Acat1*, acetyl-CoA acetyltransferase 1; *Pcsk9*, proprotein convertase subtilisin/kexin type 9; *Mogat*, monoacylglycerol acyltransferase; *Dgat1*, diacylglycerol O-acyltransferase 1; *Dgat2*, diacylglycerol O-acyltransferase 2; *Cpt1*, carnitine palmitoyltransferase 1; *Acox1*, acyl-CoA oxidase 1; Cpt2, carnitine palmitoyltransferase 2; *Pparg*, peroxisome proliferator-activated receptor gamma; *Ppara*, peroxisome proliferator-activated receptor alpha; *Pgc1*, peroxisome proliferator-activated receptor gamma coactivator 1; *Akt2*, protein kinase B2; *Cyp7a1*, cholesterol-7-alpha-hydroxylase; *Cyp8b1*, sterol 12-alpha-hydroxylase; *Cyp27a1*, sterol 27-hydroxylase; *Cyp7b1*, oxysterol 7-alpha-hydroxylase; *Cd36*, cluster of differentiation 36; Sr-bI, scavenger receptor class B type 1; *ApoA4*, apolipoprotein A-IV; *Ces2a*, carboxylesterase 2A; *Ces2c*, carboxylesterase 2C.

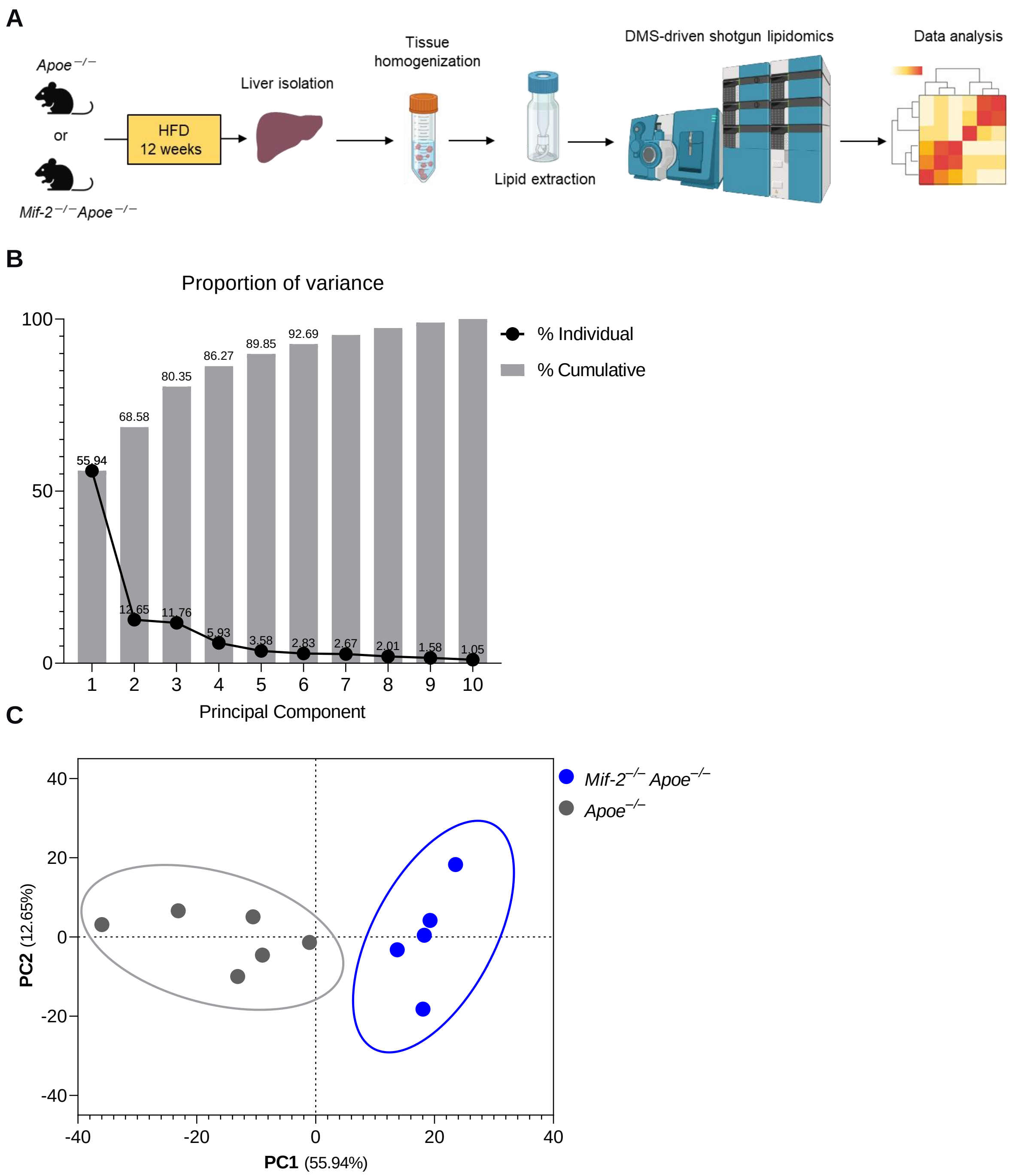

Supplementary Fig. 20: Outline and quality control of DMS-based shotgun lipidomics to investigate lipidomic differences between the livers of atherogenic *Apoe*$^{-/-}$ and *Mif-2*$^{-/-}$ *Apoe*$^{-/-}$ mice after 12 weeks of HFD. a Cartoon illustrating the lipidomics workflow (created with BioRender.com). **b** Principal component analysis (PCA) screen plot showing the proportion of variance explained by each of the first 10 principal components. **c** PCA score plot showing the separation of lipidomic profiles between *Apoe*$^{-/-}$ (n =6) (gray circles) and *Mif-2*$^{-/-}$ *Apoe*$^{-/-}$ (n=5) (blue circles) mice. (**a**) was created in BioRender. Bernhagen, L. (2025) https://BioRender.com/c59v181. Abbreviations: HFD, high-fat diet; DMS, differential mobility spectrometry.

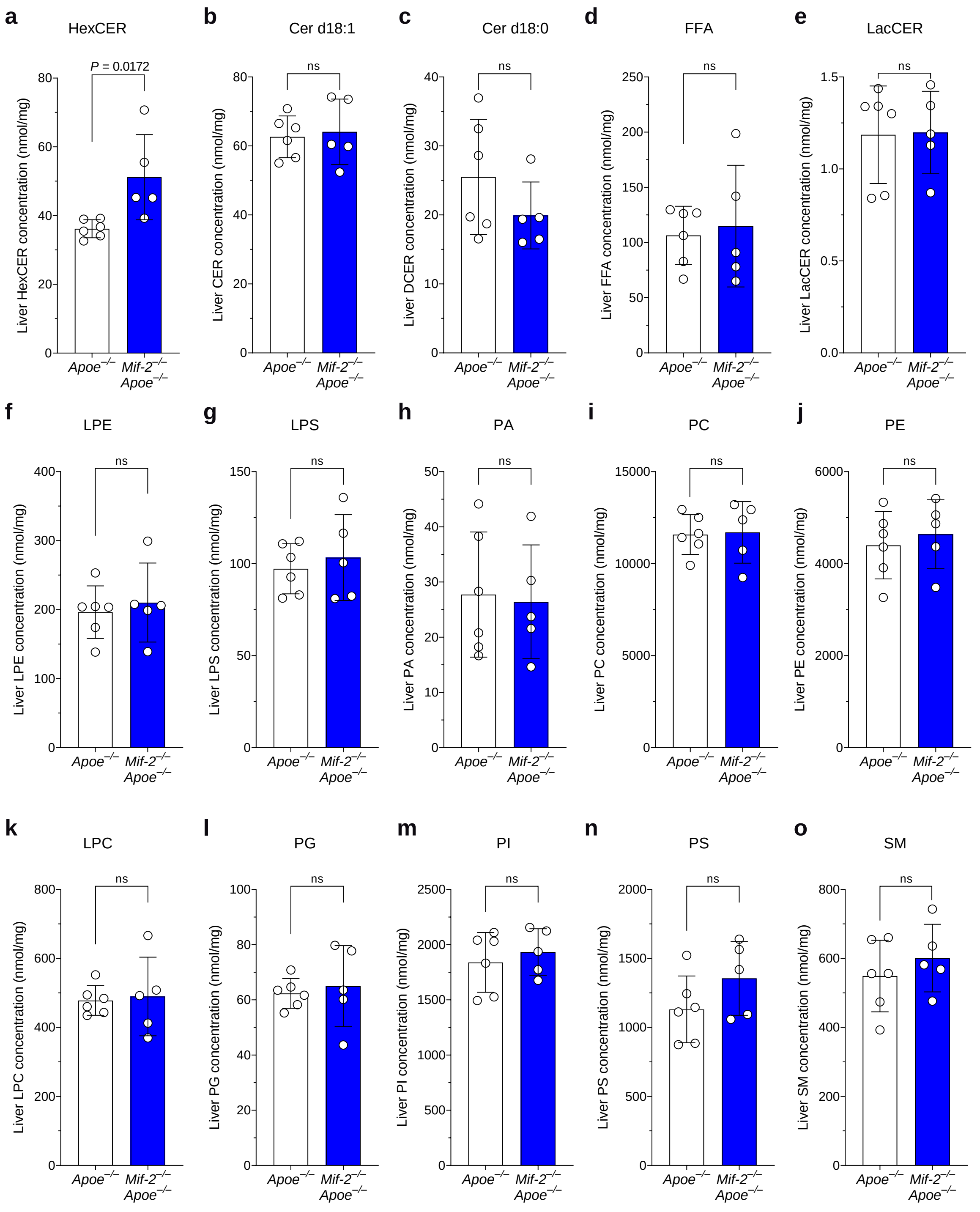

Supplementary Fig. 21: Comparison of lipid subclass concentrations in liver tissues of atherogenic *Apoe*$^{–/–}$ and *Mif-2*$^{–/–}$ *Apoe*$^{–/–}$ mice following 12 weeks of a high-fat diet (HFD). a-o Lipid concentrations (nmol/g) were quantified using DMS-driven shotgun lipidomics. White bars indicate *Apoe*$^{–/–}$ mice (n =6), and blue bars represent *Mif-2*$^{–/–}$ *Apoe*$^{–/–}$ mice (n=5). Data are expressed as means ± SD. Statistics by unpaired t-test: **P* < 0.05; ns = not significant. All values are expressed as means ± SD and statistically analyzed using unpaired two-tailed t-test. Lipid abbreviations: HexCER, hexosylceramides; Cer d18:1, ceramides; Cer d18:0, dihydroceramides; FA, free fatty acids; LacCER, lactosylceramides; LPE, lysophosphatidyl-ethanolamines; LPS, lysophosphatidylserines; PA, phosphatidic acids; PC, phosphatidylcholines; PE, phosphatidylethanolamines; LPC, lysophosphatidylcholines; PG, phosphatidylglycerols; PI, phosphatidylinositols; PS, phosphatidylserines; SM, sphingomyelins.

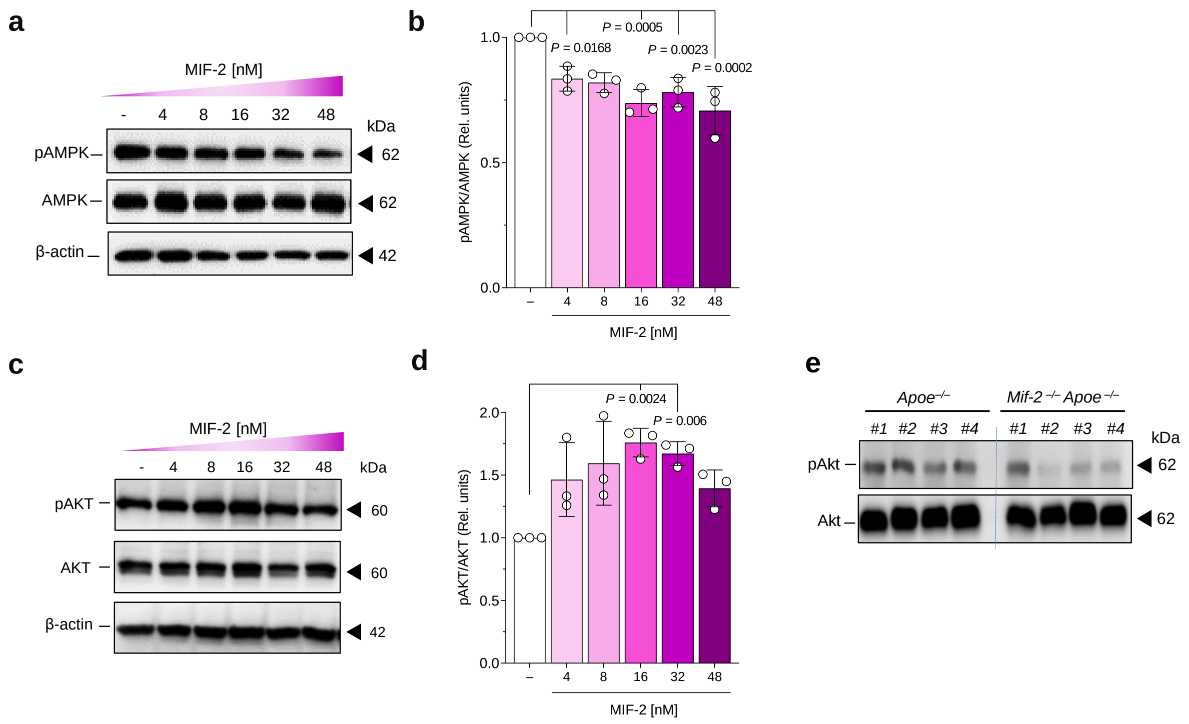

Supplementary Fig. 22: Opposite effects of MIF-2 on the AMPK and AKT signaling pathway in hepatocytes. a-b Effect of MIF-2 on AMPK activation in Huh-7 hepatocytes. Treatment with recombinant MIF-2 for 30 min; blots were developed for phospho-AMPK and AMPK (**a**, representative blot; **b**, quantification of n=3 independent experiments). β-actin was run as loading standard. **c-d** Huh-7 cells were stimulated with different concentration of rMIF-2 for 30 min, and blots were developed for phospho-AKT and AKT, β-actin is used as a loading control (**c**). Representative blot and relative quantification of n=3 independent experiments (**d**). (**e**) Western blot analysis of phospho-Akt and Akt in liver lysates from *Apoe*$^{-/-}$ and *Mif-2*$^{-/-}$*Apoe*$^{-/-}$ mice (n=4 mice per group) following 12 weeks of HFD. Values in B and D are shown as means ± SD; analysis was performed with one-way ANOVA with Dunnett's multiple comparisons test.

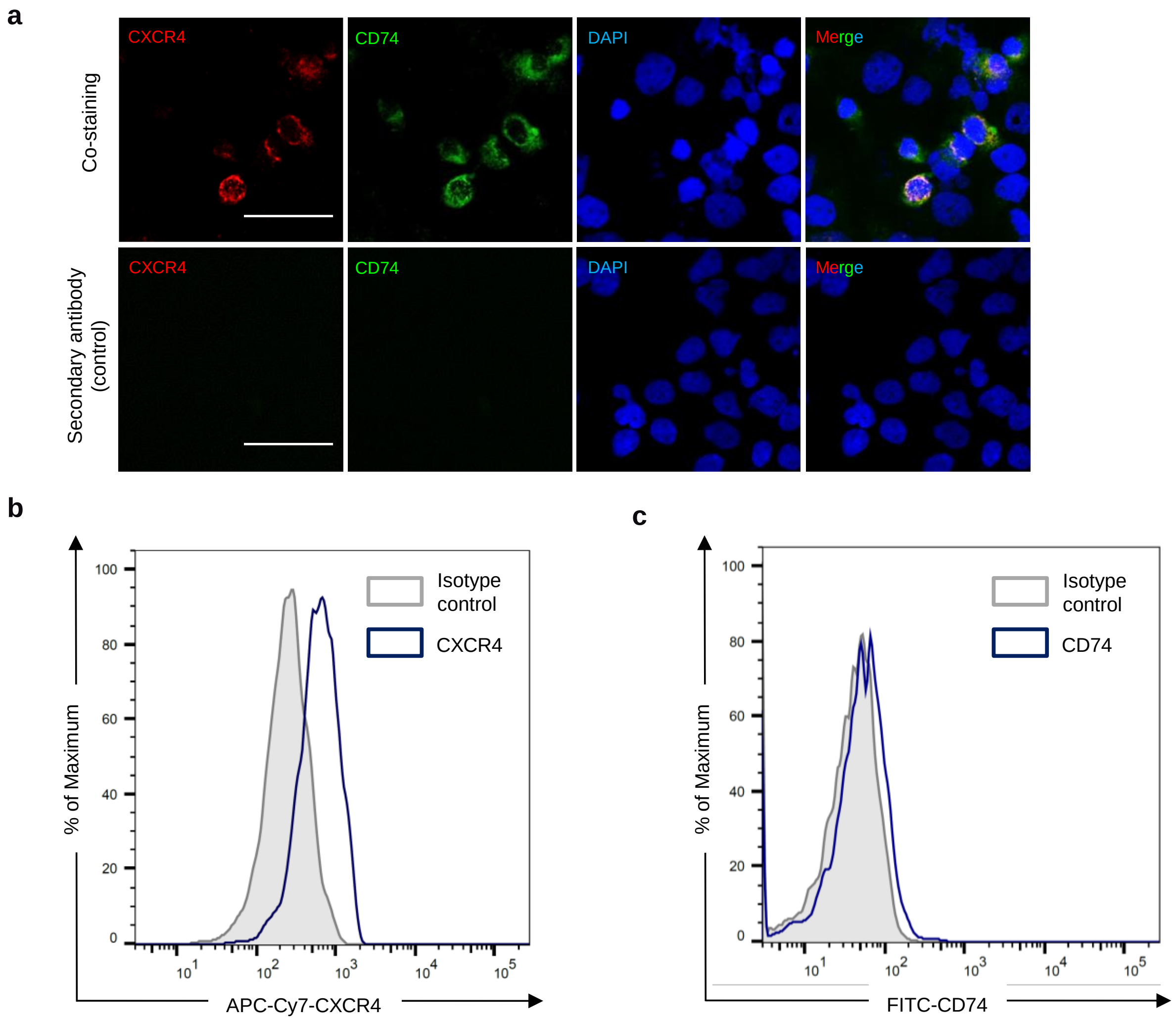

Supplementary Fig. 23: CXCR4 and CD74 are expressed in the human hepatocyte cell line Huh-7. a Colocalization of CXCR4 and CD74 in Huh-7 was visualized by immunostaining. Unstimulated cells were stained with anti-CXCR4 and anti-CD74 and corresponding fluorescently labeled secondary antibodies as indicated. Immunopositivity was detected by confocal microscopy. DAPI (blue) was used as counterstain. Scale bar: 50 µm. The secondary antibody control without primary antibody confirms specificity of the receptor expression signals. **b-c** Surface expression of CXCR4 and CD74 in Huh-7 cells was detected by flow cytometry using APC-conjugated anti-CXCR4 (**b**) and FITC-conjugated anti-CD74 (**c**) (blue) or the corresponding APC-or FITC-conjugated IgG isotype controls (grey).

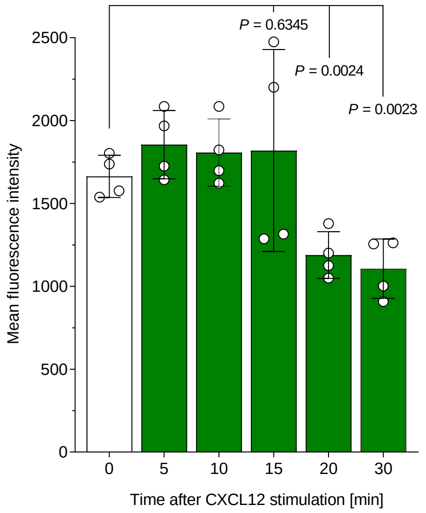

Supplementary Fig. 24: CXCL12-mediated CXCR4 internalization is delayed in the absence of CD74. Isolated splenic B cells from *Cd74*$^{-/-}$ mice were stimulated with rCXCL-12 (100 ng/ml) at 37°C for various time periods, and cell surface expression of CXCR4 was monitored by flow cytometry using APC-conjugated anti-CXCR4. n = 4 biological replicates. Data are shown as means ± SD; analysis was performed using unpaired two-tailed t-test; ns, not significant.

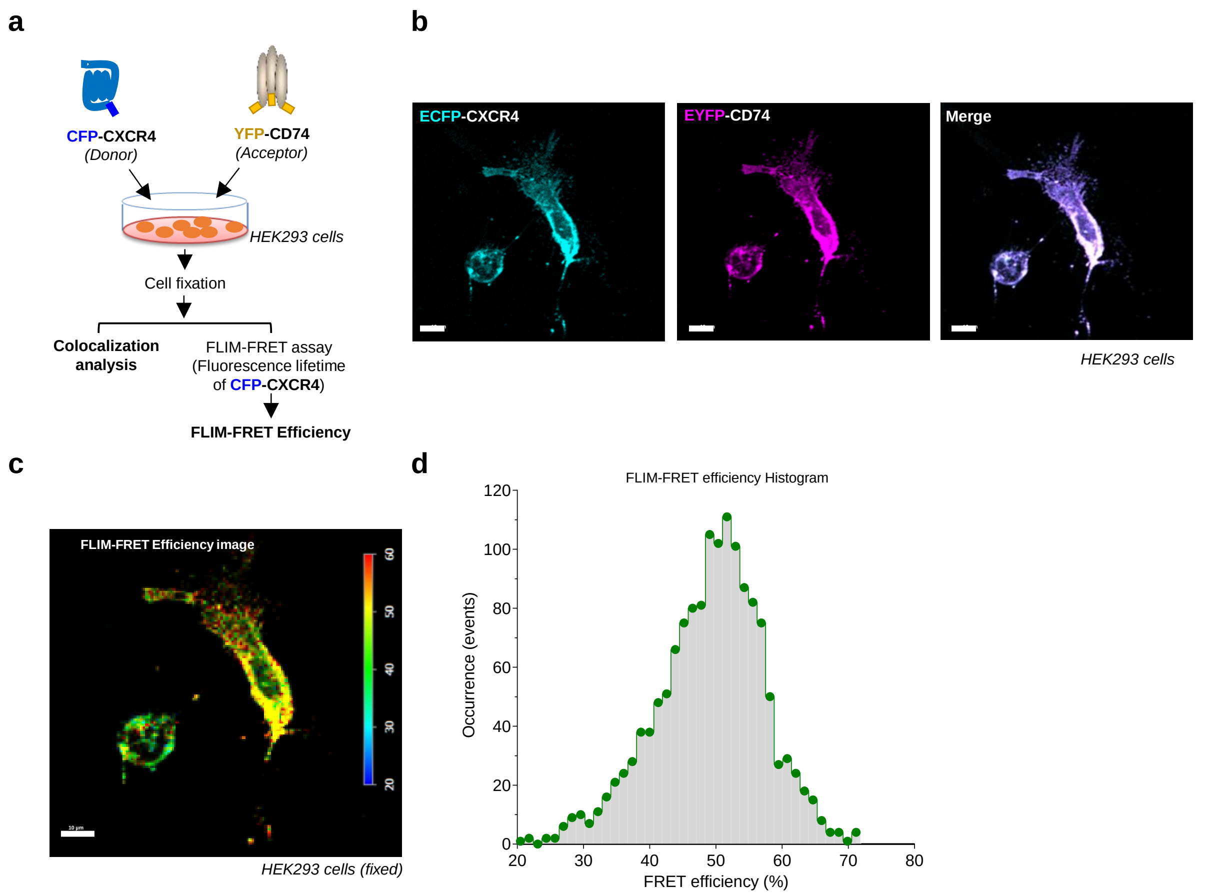

Supplementary Fig. 25: Direct interaction between CXCR4 and CD74 in HEK293 cells using multi-/two-photon fluorescence lifetime imaging microscopy. a Schematic representation of the experimental procedure. **b** Two-photon microscopy images recorded on a two/(multi)-photon laser-scanning microscopy platform (Leica TCS SP8 DIVE) showing co-localization of ECFP-CXCR4 (cyan) and EYFP-CD74 (magenta) in transfected and fixed HEK293 cells. **c-d** FLIM analysis of FRET efficiency between ECFP-CXCR4 and EYFP-CD74. FLIM image of FRET efficiency (**c**) and histogram (**d**) in fixed cells were analyzed using Leica FALCON software and showed a FRET efficiency of approximately 50%. Scale bar: 10 µm.

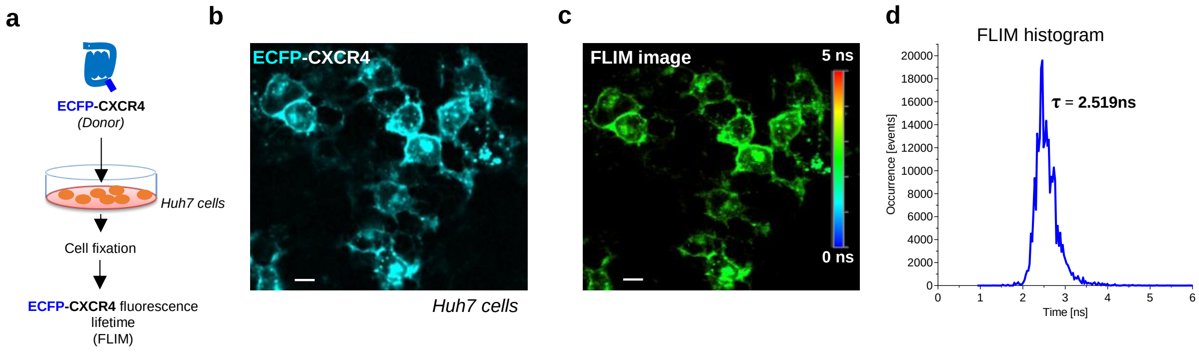

Supplementary Fig. 26: Determination of fluorescence lifetime of ECFP-CXCR4 in Huh-7 cells. a Schematic representation of the experimental procedure **(b)** Cellular distribution of ECFP-CXCR4 in Huh-7. Huh-7 cells were transfected with ECFP-tagged CXCR4 plasmid, and colocalization analysis was subsequently performed using a Leica TCSPC SP8 DIVE multiphoton laser-scanning microscope. **c-d** FLIM image and FLIM histogram of ECFP-CXCR4 transfected into Huh-7 cells. The fluorescence lifetime was determined as amplitude-weighted lifetime using Fast Lifetime Contrast (FALCON) software (τ = 2.519 ns). The FLIM image shows the amplitude-weighted lifetime (in green, corresponding to ~2.5 ns), and the FLIM histogram peaks at 2.5 ns. Scale bar, 10 µm.

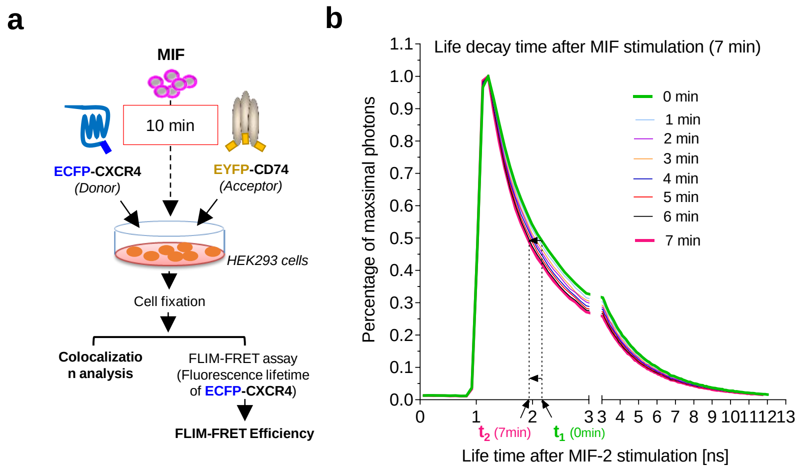

Supplementary Fig. 27: MIF also induces CXCR4/CD74 complex formation in HEK293 cells, but to a lesser extent that MIF-2. a Schematic representation of the experimental procedure. Same as in **Figure 6F**, except that transfected HEK293 cells were stimulated with MIF (8 nM) (rather than MIF-2) for 10 min at 37°C. **b** Normalized fluorescence lifetime decay curves of ECFP-CXCR4 were evaluated in live imaging mode at one-minute intervals during a 10-minute exposure to MIF. MIF stimulation was associated with a slight decrease in ECFP-CXCR4 lifetime (from ~ t_1=2.2 ns to ~ t_2=1.85 ns). The experiment shown is one out of 2 independent experiments performed with similar outcome.

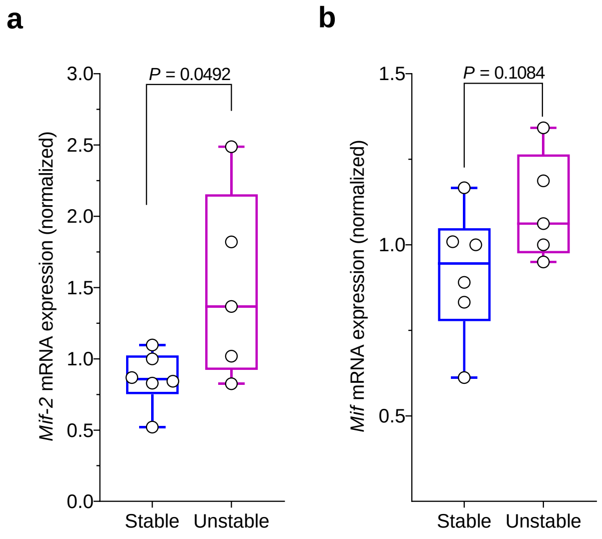

Supplementary Fig. 28: Re-analysis of mRNA expression levels of *MIF-2* and *MIF* in stable and ruptured human atheromatous plaques. a-b *MIF-2/D-DT* and *MIF* gene expression in stable *versus* unstable plaque tissue. Data are from the GSE41571 dataset downloaded from the gene expression omnibus (GEO) (Lee et al, 2013; [3]). In brief, 5 ruptured and 6 stable human atheromatous plaques from carotid endarterectomy specimens were laser-microdissected and RNA was subsequently isolated. Transcripts were then profiled using Affymetrix HG-U133 plus 2.0 GeneChip arrays. Normalized *MIF-2/D-DT* (**a**) and *MIF* (**b**) gene expression from transcriptional profiling is shown as box plots. Statistical significance was analyzed by unpaired t-test. Data are shown as means ± SD.

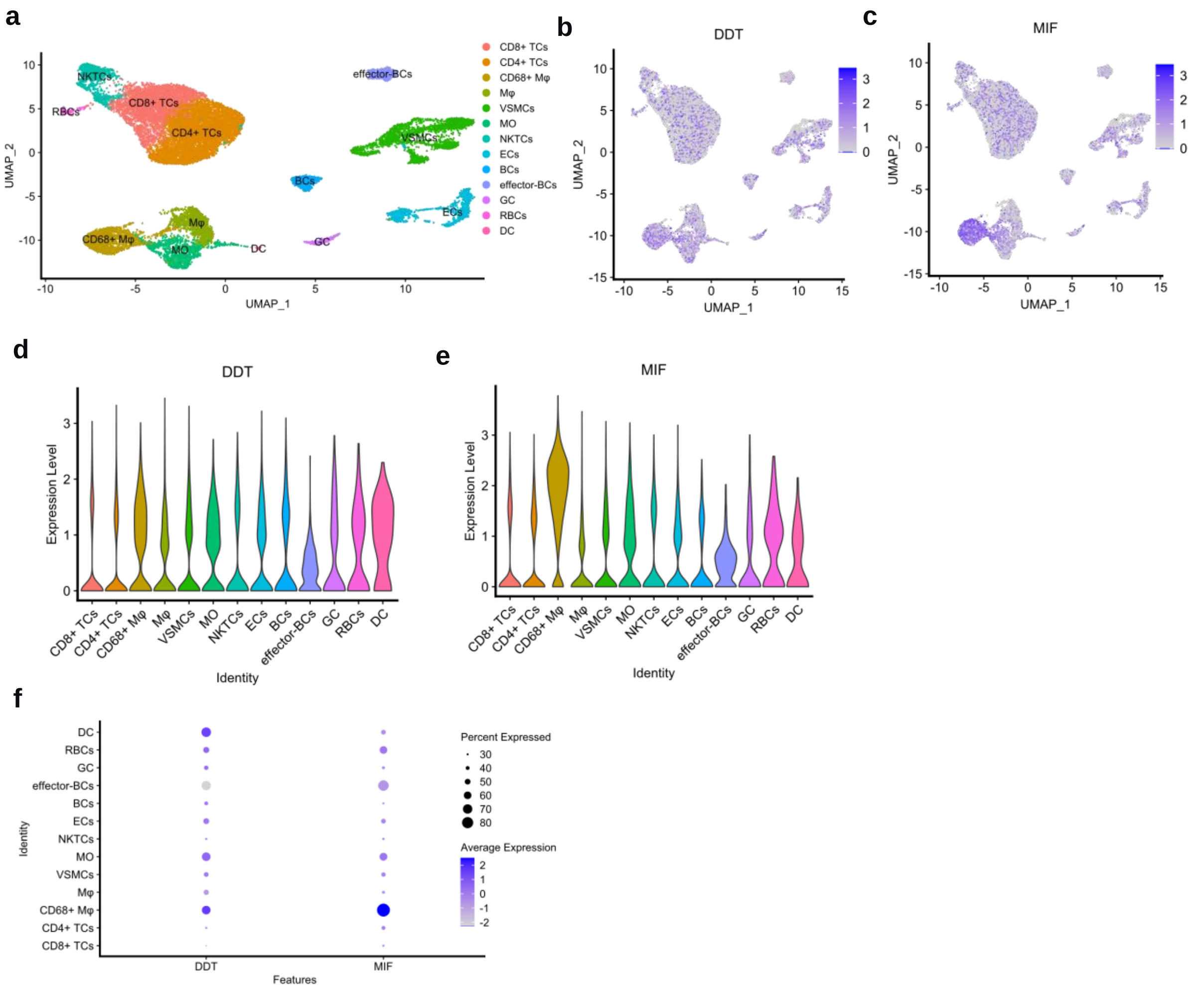

Supplementary Fig. 29: Re-analysis of single-cell RNAseq data of human atherosclerotic plaques reveals similar expression pattern of MIF-2 and MIF in atherogenic cell populations. Re-analysis of the single-cell RNAseq data set GSE159677 obtained from human atherosclerotic plaques. Downloaded from the gene expression omnibus (GEO) (reference: Alsaigh et al, 2022; [4]). **a** Visualization of single cell clusters by Uniform Manifold Approximation and Projection (UMAP) of atherosclerotic core (AC) of three human CEA patients represented by different colors. **b-c** Feature plots showing the expression of MIF-2 (designated as DDT in the data set) (**b**) and MIF (**c**) in different single cell clusters. **d-e** Violin plots showing the expression of MIF-2 (designated as DDT in the data set) (**d**) and MIF (**e**) in different atherogenic cell populations. **f** Dot plot showing the comparison between the expression of both MIF and MIF-2. Dot size represent the percentage (%) of expression, and color intensity indicates the average expression level. BCs, B cells; TCs, T cells; NKTCs, natural killer T cells; RBCs, red blood cells; VSMCs, vascular smooth muscle cells; ECs, endothelial cells; GC, granulocytes; DC, dendritic cells; MO, monocytes; Mφ, macrophages.

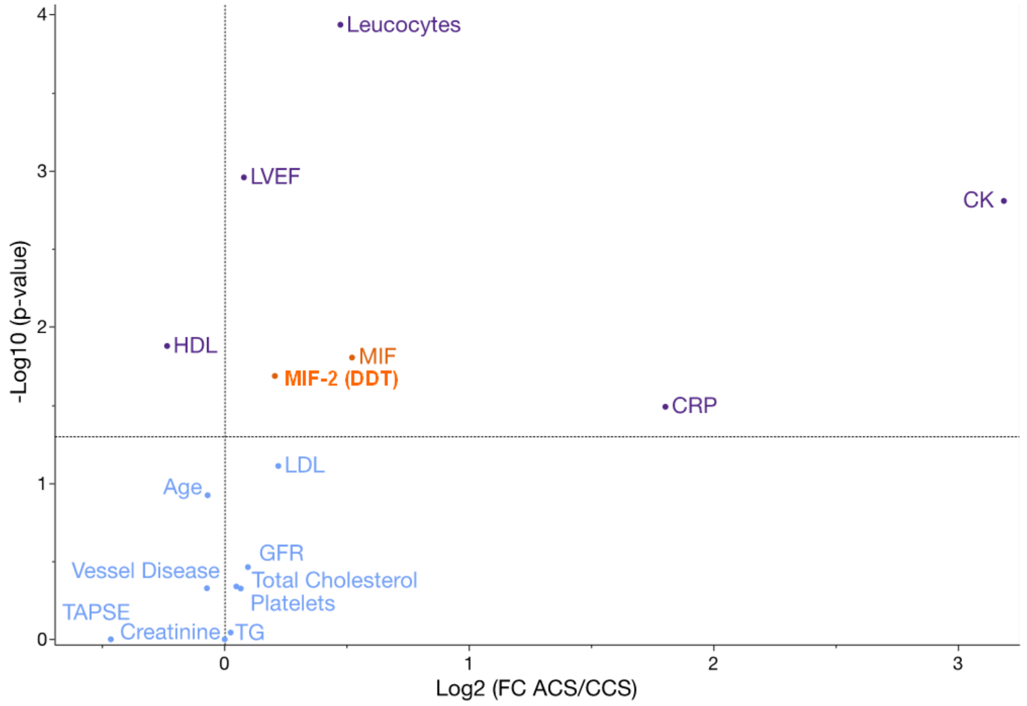

Supplementary Fig. 30: Volcano plot of the gene expression of *MIF* and *MIF-2* together with significantly differentiated clinical parameters in CAD patients. The x-axis displays log2–transformed fold change between patients with troponin+/ACS and troponin-/CCS. The y-axis shows the -log10–transformed *P* value of student´s t-test. The horizontal line highlights significant alterations (*P*<0.05). Values >1 indicate an increase in patients with ACS; values <1 indicate a decrease in contrast to patients with CCS. LVEF, left ventricular ejection fraction; CRP, C-reactive protein; GFR, glomerular filtration rate; CK, creatine kinase; HDL, high density lipoprotein; LDL, low density lipoprotein; TG, triglyceride; TAPSE, tricuspid annular plane systolic excursion.

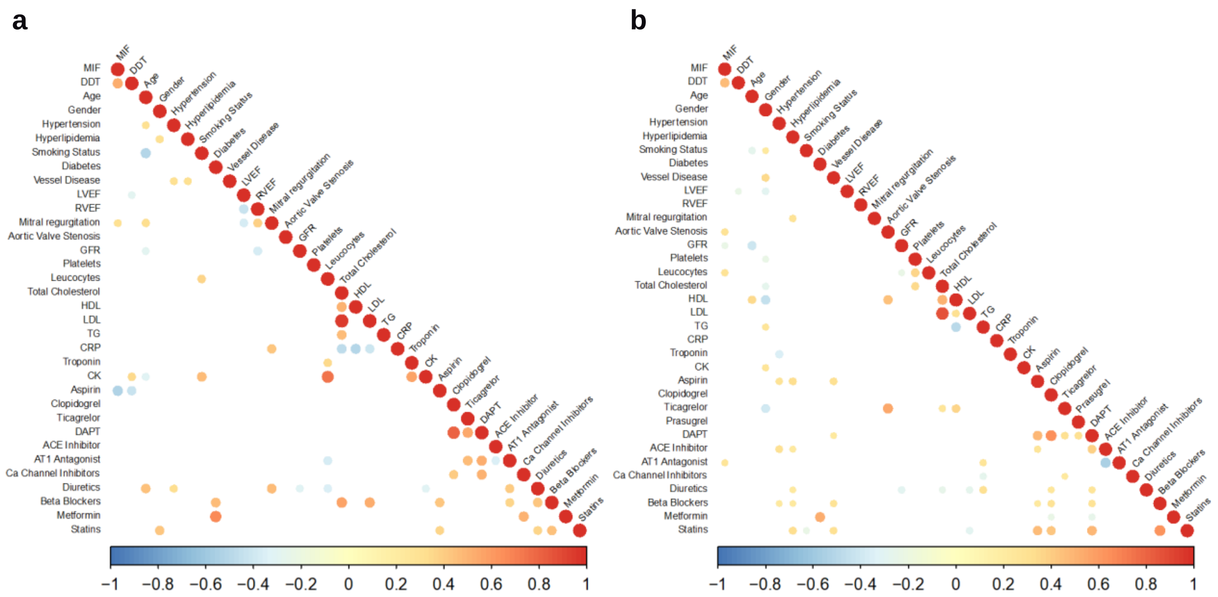

Supplementary Fig. 31: Correlation analysis of MIF and MIF-2 (designated as DDT in the data set) expression with baseline clinical parameters, including cardiovascular risk factors, laboratory parameters, and concomitant medication in ACS patients (**a**) and CCS patients (**b**). Significant ($P<0.05$) coefficients of Pearson correlation (rρ) are highlighted and colored accordingly. LVEF, left ventricular ejection fraction; CVRF, cardiovascular risk factors; CRP, C-reactive protein; GFR, glomerular filtration rate; CK, creatine kinase; HDL, high density lipoprotein; LDL, low density lipoprotein; ASA, acetylsalicylic acid; P2Y12, adenosine diphosphate receptor P2Y12 inhibitors; TG, triglyceride; ACE, angiotensin-converting enzyme; AT1, type I angiotensin.

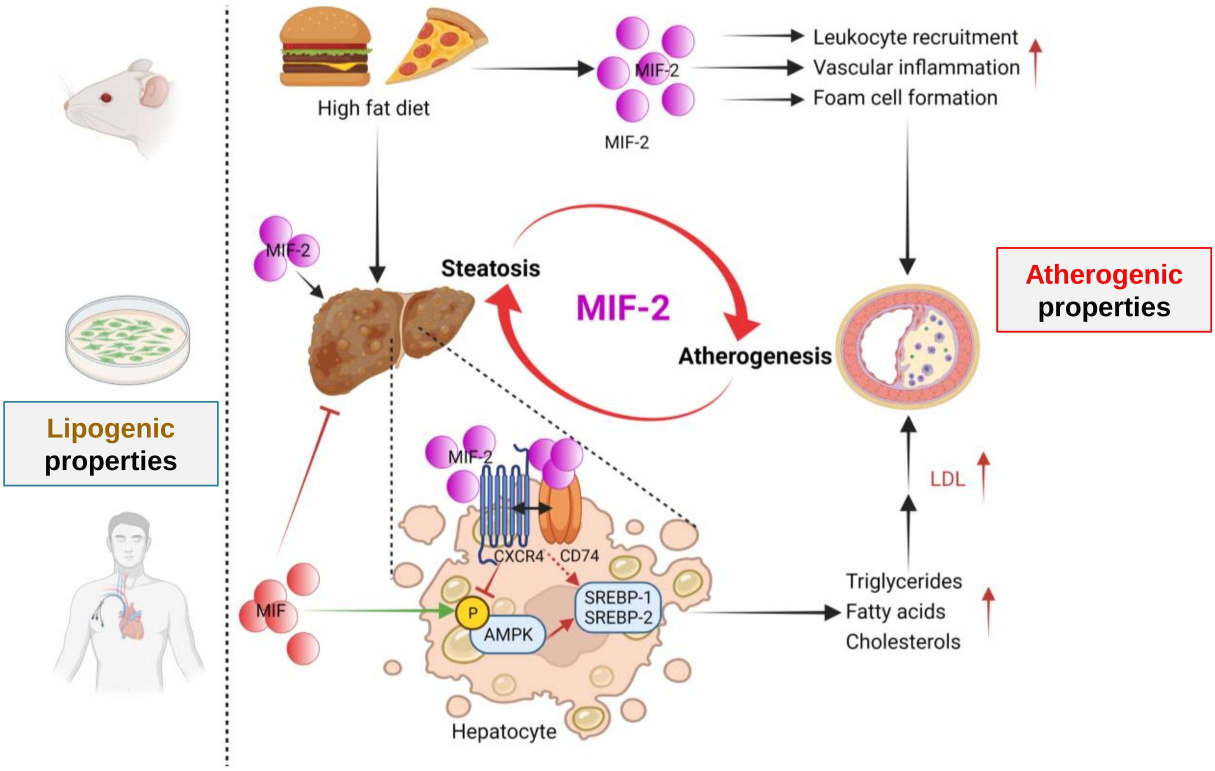

Supplementary Fig. 32: The role of MIF-2 in advanced atherosclerosis and hepatic lipogenesis. The cartoon depicts the dual role of MIF-2, promoting both an atherogenic vascular and a hepatic/lipid phenotype. MIF plays a crucial role in advanced atherosclerosis, linking both atherogenesis and steatosis through its dual atherogenic and lipogenic properties. Its atherogenic actions involve promoting the recruitment of leukocytes, vascular inflammation, and foam cell formation (top right), which contribute to the development of atherosclerotic lesions (right). Simultaneously, its lipogenic properties affect hepatic lipogenesis through CXCR4 and CD74 receptors (left), which trigger SREBPs activity (bottom), leading to increased synthesis of triglycerides, fatty acids, and cholesterol (bottom right). This, in turn, exacerbates the progression of atherosclerosis. Abbreviations: MIF-2, macrophage migration inhibitory factor-2 (also known as D-dopachrome tautomerase, D-DT); AMPK, AMP-activated protein kinase (5'-adenosine monophosphate-activated protein kinase); SREBP1/2 sterol-regulatory element-binding proteins 1 and 2; LDL, low-density lipoprotein; CD74, cluster of differentiation 74 (also referred to as HLA class II histocompatibility antigen gamma chain); CXCR4, C-X-C motif chemokine receptor 4. The scheme was created in BioRender. Bernhagen, L. (2025) https://BioRender.com/l02i034.

References cited in Supplementary File

1. Wirka RC, Wagh D, Paik DT, Pjanic M, Nguyen T, Miller CL, Kundu R, Nagao M, Coller J, Koyano TK, Fong R, Woo YJ, Liu B, Montgomery SB, Wu JC, Zhu K, Chang R, Alamprese M, Tallquist MD, Kim JB, Quertermous T. Atheroprotective roles of smooth muscle cell phenotypic modulation and the TCF21 disease gene as revealed by single-cell analysis. *Nat Med* **25**, 1280-1289 (2019).
2. Pan H, Xue C, Auerbach BJ, Fan J, Bashore AC, Cui J, Yang DY, Trignano SB, Liu W, Shi J, Ihuegbu CO, Bush EC, Worley J, Vlahos L, Laise P, Solomon RA, Connolly ES, Califano A, Sims PA, Zhang H, Li M, Reilly MP. Single-cell genomics reveals a novel cell state during smooth muscle cell phenotypic switching and potential therapeutic targets for atherosclerosis in mouse and human. *Circulation* **142**, 2060-2075 (2020).
3. Lee K, Santibanez-Koref M, Polvikoski T, Birchall D, Mendelow AD, Keavney B. Increased expression of fatty acid binding protein 4 and leptin in resident macrophages characterises atherosclerotic plaque rupture. *Atherosclerosis* **226**, 74-81 (2013).
4. Alsaigh T, Evans D, Frankel D, Torkamani A. Decoding the transcriptome of calcified atherosclerotic plaque at single-cell resolution. *Commun Biol* **5**, 1084 (2022).
