# Supplementary material for: An atypical atherogenic chemokine that promotes advanced atherosclerosis and hepatic lipogenesis
Source: Nat Commun. 2025 Mar 7;16:2297. doi: 10.1038/s41467-025-57540-z (PMC11889166; doi:10.1038/s41467-025-57540-z)
Supplement: Supplementary file 2 — Reporting Summary [file 41467_2025_57540_MOESM2_ESM.pdf]

nature portfolio

Corresponding author(s): Jürgen Bernhagen & Omar El Bounkari

Last updated by author(s): 15/01/25

Reporting Summary

Nature Portfolio wishes to improve the reproducibility of the work that we publish. This form provides structure for consistency and transparency in reporting. For further information on Nature Portfolio policies, see our Editorial Policies and the Editorial Policy Checklist.

Statistics

For all statistical analyses, confirm that the following items are present in the figure legend, table legend, main text, or Methods section.

n/a	Confirmed	
☐	☒	The exact sample size (n) for each experimental group/condition, given as a discrete number and unit of measurement
☐	☒	A statement on whether measurements were taken from distinct samples or whether the same sample was measured repeatedly
☐	☒	The statistical test(s) used AND whether they are one- or two-sided<br>*Only common tests should be described solely by name; describe more complex techniques in the Methods section.*
☐	☒	A description of all covariates tested
☐	☒	A description of any assumptions or corrections, such as tests of normality and adjustment for multiple comparisons
☐	☒	A full description of the statistical parameters including central tendency (e.g. means) or other basic estimates (e.g. regression coefficient) AND variation (e.g. standard deviation) or associated estimates of uncertainty (e.g. confidence intervals)
☐	☒	For null hypothesis testing, the test statistic (e.g. F, t, r) with confidence intervals, effect sizes, degrees of freedom and P value noted<br>*Give P values as exact values whenever suitable.*
☒	☐	For Bayesian analysis, information on the choice of priors and Markov chain Monte Carlo settings
☒	☐	For hierarchical and complex designs, identification of the appropriate level for tests and full reporting of outcomes
☒	☐	Estimates of effect sizes (e.g. Cohen's d, Pearson's r), indicating how they were calculated

Our web collection on statistics for biologists contains articles on many of the points above.

Software and code

Policy information about availability of computer code

Data collection	Flow Cytometry: BD FACSVerse™ Flow Cytometer (BD Biosciences); BD FACSuite™ (FCS 3.0/2.0) software (Becton Dickinson).<br>PCR and RT-qPCR: Biometra TRIO 48 Touch Thermocycler (Analytik Jena GmbH); NanoDrop One Microvolume UV-Vis Spectrophotometer (Thermo Fisher Scientific); Rotor-Gene 6000 (Qiagen, Hilden, Germany); Rotor-Gene 6000 Series v.1.7 software (Corbett); TissueLyser LT (Qiagen); TC20™ Automated Cell Counter (Bio-Rad Laboratories GmbH).<br>IHC (Immunohistochemistry): Cryotome Leica CM1950 Platform (Leica Microsystems CMS GmbH); Leica Dissection Microscope (Leica Microsystems CMS GmbH); Embedding Machine STP 120 (Leica Microsystems CMS GmbH).<br>Fluorescence Microscopy: DMi8-Life Cell Imaging System (Leica Microsystems, Mannheim, Germany); Confocal Microscope (Carl Zeiss AG).<br>Chemotaxis and Flow Adhesion: Manual Tracking, Chemotaxis, and Migration Tools (Ibidi GmbH); Ibidi Pump System (Ibidi GmbH).<br>Western Blot/Dot Blot: Odyssey® Fc Imager and Image Studio™ v.5.2 software (LI-COR Biosciences).<br>Fluorescence Spectroscopy and Circular Dichroism (CD): JASCO J-715 Spectropolarimeter; JASCO FP-6500 Fluorescence Spectrophotometer; OriginPro 2016 (OriginLab Corporation).<br>Multiplex-Bead-Array ELISA: Luminex™ 200™ Instrument System (Luminex Corporation); xPONENT v.3.1 software (Luminex Corporation); ProcartaPlex Analysis Software (Thermo Fisher, Invitrogen).<br>Two-Photon Microscopy (2PM) / FLIM-FRET: Leica TCS SP8 DIVE Multiphoton Microscope (Leica Microsystems CMS GmbH); LAS X software, Leica FALCON, and Leica LIGHTNING deconvolution algorithms (Leica Microsystems).<br>ELISA: EnSpire 2300 Multimode Plate Reader (PerkinElmer LAS GmbH).<br>Lipidomics: Precellys 24 Homogenizer (PEQLAB Biotechnology GmbH, Germany); SCIEX Exion UHPLC System coupled to a SCIEX QTRAP 6500+ Mass Spectrometer.<br>Plasma Cholesterol and Triglycerides Concentration: Fast Protein Liquid Chromatography (FPLC, Cytiva).<br>Bulk RNA Sequencing: Bulk RNA sequencing from frozen liver sections embedded in OCT was performed by BGI Tech Solutions Co., Limited

	(Hong Kong, China). For RNA sequencing from liver lysates, libraries were prepared using the Prime-Seq protocol and processed with the zUMIs pipeline following a standard workflow as described in references 110 and 111. Bioanalyzer (Agilent Technologies); Qubit DNA HS Assay Kits and Qubit 4.0 Fluorometer (Invitrogen); Galaxy Platform (https://usegalaxy.org/) using HISAT2, FastQC, and DESeq2 v.1.44.0; clusterProfiler package (v.4.12.0) and EnhancedVolcano package (v.1.22.0).
Data analysis	GraphPad Prism v.9 (GraphPad Inc.); FlowJo v.10 (Tree Star Inc.); ImageJ v.1.53 (NIH); FACSuite (FCS 3.0/2.0) software (BD); Manual tracking, chemotaxis, and migration tools (Ibidi GmbH); ProcartaPlex Analysis software (Thermofisher Scientific); Rotor-Gene 6000 Series 1.7 software (Corbett reesarch); xPONENT v. 3.1 software (Luminex Corporation); LAS-X and Leica FALCON software (Leica Microsystems), HADDOCK 2.4 (HADDOCK Webserver); Matlab2022a (MathWorks); Shotgun Lipidomics Assistant software (SLA.v1.5; https://github.com/syjgino/SLA/tree/v1.5-keyV4). RNA-seq data analysis was performed on the Galaxy platform (https://usegalaxy.org/) using HISAT2, FastQC, and DESeq2 v.1.44.0. Gene ontology analysis was conducted using clusterProfiler package (4.12.0). Deconvolution was carried out using Leica LIGHTNING deconvolution algorithms in LAS X and Leica FALCON software. Molecular interactions of MIF-2/CXCR4, MIF/CXCR4, and MIF-2/msR4M-L1 complexes were analyzed using HADDOCK docking simulations (HADDOCK Webserver 2.4; https://rascar.science.uu.nl/haddock2.4/) and data Matlab2022a (MathWorks).

For manuscripts utilizing custom algorithms or software that are central to the research but not yet described in published literature, software must be made available to editors and reviewers. We strongly encourage code deposition in a community repository (e.g. GitHub). See the Nature Portfolio guidelines for submitting code & software for further information.

Data

Policy information about availability of data

All manuscripts must include a data availability statement. This statement should provide the following information, where applicable:

- Accession codes, unique identifiers, or web links for publicly available datasets
- A description of any restrictions on data availability
- For clinical datasets or third party data, please ensure that the statement adheres to our policy

All data supporting the findings of this study are included within the manuscript and Supplementary Information files. Source data for the main and supplementary figures are provided in a Source Data Excel file underlaying Fig 1b-h, Fig 1j-m, Fig 2a-c, Fig 2e-g, Fig 2i, Fig 3c-f, Fig 3h, Fig 3i, Fig 3k-l, Fig 3n, Fig 4b-g, Fig 4i-l, Fig 5b, Fig 5d-g, Fig 5i, Fig 5k-l, Fig 6a-d, Fig 7a-b, Fig 7d, Fig 7i, Fig 7l, Fig 7n, Fig 7q, Fig 7r, Fig 8b-f, Fig S1b-c, Fig S2c-d, Fig S3a-d, Fig S4a-c, Fig S5a-b, Fig S1b-c, Fig S8b, Fig S8d, Fig S9b, Fig S12a-b, Fig S12d, Fig S13a-d, Fig S15a-b, Fig S17b-c, Fig S19a-d1, Fig S20b-c, Fig S21a-0, Fig S22a-d, Fig S24, Fig S25d, Fig S26d, Fig S27b, and Fig S28a-b. Additionally, bulk RNA sequencing data of the OCT-embedded frozen liver tissue sections of Apoe-/- versus Mif-2-/-Apoe-/- after 12 weeks on a high-fat diet (HFD) have been deposited in the Gene Expression Omnibus (GEO) and are accessible under accession code GSE281285 (https://www.ncbi.nlm.nih.gov/geo/query/acc.cgi?acc=GSE281285). Additionally, RNA-seq data from whole liver lysates can be accessed under GEO accession number GSE287230 (https://www.ncbi.nlm.nih.gov/geo/query/acc.cgi?acc=GSE287230).The lipidomics data have been referenced by MetaboLights under accession number REQ20250117208170 and are accessible via the following link: https://www.ebi.ac.uk/metabolights/editor/study/REQ20250117208170.

Research involving human participants, their data, or biological material

Policy information about studies with human participants or human data. See also policy information about sex, gender (identity/presentation), and sexual orientation and race, ethnicity and racism.

Reporting on sex and gender	Patients characteristics including age and gender are depicted supplementary table S3
Reporting on race, ethnicity, or other socially relevant groupings	None
Population characteristics	Provided in Supplementary table S3
Recruitment	Recruitment criteria was described in reference 110, 111, 121 and 122.
Ethics oversight	The study for CEA patients received approval from the local ethics committee at the Medical Faculty of the Klinikum rechts der Isar, Technical University Munich (ethics approval 2799-10), as previously detailed in references 118 and 119. CAD patients were admitted to the Department of Cardiology at the University Hospital of Tübingen, Germany, as described in references 121 and 122.

Note that full information on the approval of the study protocol must also be provided in the manuscript.

Field-specific reporting

Please select the one below that is the best fit for your research. If you are not sure, read the appropriate sections before making your selection.

☒ Life sciences ☐ Behavioural & social sciences ☐ Ecological, evolutionary & environmental sciences

For a reference copy of the document with all sections, see nature.com/documents/nr-reporting-summary-flat.pdf

Life sciences study design

All studies must disclose on these points even when the disclosure is negative.

Sample size	Sample size calculations were not conducted in this study. For biochemical assays, the typical sample size was n = 3 (biologically independent experiments), consistent with standard practice in biochemical studies and our prior experience (e.g. Doring et al. Nat Cardiovasc Res, 2024;

	Burger-Kentischer et al. Circulation, 2002 ; Qi et al. J Clin Invest, 2009; Klasen et al., J. Immunol., 2014; Alampour-Rajabi et al., FASEB J., 2015; Kontos et al., Nat. Commun., 2020; Tas et al., Nat. Commun., 2022). The in vivo atherosclerosis experiments were conducted with 11-12 mice per group. Sample sizes for experiments involving tissue specimens from human atherosclerotic CEA patients (and their controls) were determined based on prior experience with the same cohort/biobank, similar to our previous work (e.g. Merckelbach et al., Thromb Haemost 2018; Pelisek et al., J Clin Med 2019; Kontos et al., Nat. Commun., 2020; Sharifi et al., Circ. Res. 2023; Wang et al., Cardiovasc. Res. 2024). Plasma MIF-2 levels were measured in CAD patients (N = 149), with CAD severity assessed through coronary angiography, further categorizing patients into acute coronary syndrome (ACS) (n = 47) and chronic coronary syndrome (CCS) (n = 85) subgroups.
Data exclusions	Data were excluded only in rare cases due to major technical issues. In downstream analysis of the Bulk RNA-seq data, to reduce noise from lowly expressed genes, we retained only genes with counts of at least one in at least three samples within either the Mif-2–/–Apoe–/– or Apoe–/– groups. Additionally, the top 10 most highly expressed genes were excluded to prevent skewing in downstream analyses.
Replication	The number of independently conducted experiments is indicated in the legends of the figures and supplementary figures throughout the manuscript, with all replications completed successfully.
Randomization	For pharmacological blockade, mice were randomly assigned to groups and were age- and gender-matched littermates. A random allocation of samples/specimens was not relevant in the other parts of the study.
Blinding	Investigators were not blinded to group allocation during data collection. For data analysis, investigators were blinded, and/or analysis guided by software-based analysis (e.g. ACSuite (FCS 3.0/2.0) software (BD), Ibidi migration and tracking tools, ProcartaPlex Analysis, xPONENT v. 3.1 software from Luminex, Leica LAS-X, FALCON, and LIGHTNING software, shotgun lipidomics assistant software SLA.v1.5) as indicated in methods and figure legends. RNA-seq data analysis was performed by HISAT2, FastQC, and DESeq2 v.1.44.0 software.

Reporting for specific materials, systems and methods

We require information from authors about some types of materials, experimental systems and methods used in many studies. Here, indicate whether each material, system or method listed is relevant to your study. If you are not sure if a list item applies to your research, read the appropriate section before selecting a response.

Materials & experimental systems

n/a	Involved in the study
☐	☒ Antibodies
☐	☒ Eukaryotic cell lines
☐	☐ Palaeontology and archaeology
☐	☒ Animals and other organisms
☐	☐ Clinical data
☐	☐ Dual use research of concern
☐	☐ Plants

Methods

n/a	Involved in the study
☒	☐ ChIP-seq
☐	☒ Flow cytometry
☐	☐ MRI-based neuroimaging

Antibodies

Antibodies used

WB:
Anti-SREBP-1 mouse monoclonal antibody 1:500 (Santa Cruz Biotechnology, Cat# sc-17755)
Anti-SREBP-2 mouse monoclonal antibody 1:1000 (BD Biosciences, Cat# 557037)
Anti-FASN mouse monoclonal antibody 1:500 (Santa Cruz Biotechnology, Cat# sc-55580)
Anti-LDLR mouse monoclonal antibody 1:500 (Santa Cruz Biotechnology, Cat# sc-18823)
Anti-D-DT rabbit polyclonal antibody 1:1000, generated by R. Bucala and colleagues in the Bucala lab (Yale University) as reported in e.g.: Merk et al., The D-dopachrome tautomerase (DDT) gene product is a cytokine and functional homolog of macrophage migration inhibitory factor (MIF). PNAS 108, E577-585, 2011; Ma et al., Cardiomyocyte D-dopachrome tautomerase protects against heart failure. JCI Insight 4:e128900, 2019.
Anti-AMPKα rabbit monoclonal antibody 1:1000 (Cell Signaling Technology, Cat# 5832S)
Anti-phospho-AMPKα rabbit monoclonal antibody 1:1000 (Cell Signaling Technology, Cat# 2535)
anti-Akt (pan) (C67E7) Rabbit mAb 1:1000 (Cell Signaling, cat# 4691)
anti-phospho-Akt (Ser473) antibody 1:1000 (Cell Signaling, Cat#9271)
Anti-β-actin monoclonal antibody 1:1000 (Santa Cruz Biotechnology, cat: sc-47778)
Goat anti-mouse HRP-conjugated antibody 1:10000 (Abcam, Cat# ab6789)
Goat anti-rabbit HRP-conjugated antibody 1:25000 (GE Healthcare Cat# NA934V)
IF:
Anti-CXCR4 rabbit polyclonal antibody (Invitrogen, Cat# PA3-305)
Anti-CD74 mouse monoclonal antibody (Santa Cruz Biotechnology Cat# sc-6262)
ElisaAlexa Fluor 647-conjugated donkey anti-rat IgG (H+L) (Jackson ImmunoResearch Europe Ltd., Cat# 712-606-153)
Alexa Fluor 488-conjugated goat anti-rat IgG (H+L) (Invitrogen; Cat# A-11006)
Alexa Fluor 555-conjugated goat anti-rabbit IgG (H+L) (Invitrogen, Cat# A-21429)
IHC:
Anti-CD68 rat monoclonal antibody 1:100 (Bio-Rad Laboratories, Cat# MCA1957GA)
Cy5-conjugated donkey Anti-Rat IgG (H+L) (Jackson ImmunoResearch Europe Ltd., Cat# 712-175-153)

Internalization:
Mouse PE-conjugated anti-CXCR4 antibody (R&D systems, Cat# FAB21651P)
Rat PE-conjugated anti-mouse IgG2B Antibody (R&D systems, Cat# F0132)
PLA:
mouse anti-cMyc (9E10) mAb 1:200 (Santa Cruz, cat# sc-40)
rabbit anti-DYKDDK Tag (D6W5B) mAb 1:200 (anti-FLAG, Cell Signaling Technologies, cat# 14793)
LDL uptake:
anti-human CD74 (LN2) (Santa Cruz, Cat# sc-6262)
IgG1 κ light chain antibody (2B6) (Santa Cruz, Cat# sc-69914)
Flow Cytometry:
V450-conjugated rat anti-mouse CD45 (BD Biosciences Cat# 560501)
FITC-conjugated anti-mouse CD3 (Miltenyi Biotec, Cat# 130-119-758)
APC/Cy7-conjugated anti-mouse CD19 (BioLegend Cat# 115530)
PE-conjugated anti-mouse CD11c (BioLegend, Cat# 117308)
PE/Cy7-conjugated anti-mouse/human CD11b Antibody (BioLegend, Cat# 101216)
APC-conjugated anti-mouse Ly-6C (BioLegend, Cat# 128016)
PerCP-conjugated anti-mouse Ly-6G (BioLegend, Cat# 127654)
APC/Cy7-cponjugated anti-human CD184 (CXCR4) (Biolegend, Cat# 306527)
APC/C7-conjugated mouse anti-human IgG2a, κ Isotype Ctrl (Biolegend, Cat# 400229)
FITC-conjugated mouse anti-human CD74 (BD Pharmingen, Cat# 555540)
FITC-conjugated anti-human IgG2 (isotype control) (BD Pharmingen, Cat# 560952)

Validation

• Anti-SREBP-1 mouse monoclonal antibody (Santa Cruz Biotechnology, Cat# sc-17755)
Reactivity: Human; Host species: Mouse; Application: WB/IP/IHC/Elisa
https://www.antibodyregistry.org/AB_628283
• Anti-SREBP-2 mouse monoclonal antibody (BD Biosciences, Cat# 557037)
Reactivity: Human; Host species: Mouse; Application: WB/IP
https://www.bdbiosciences.com/en-fi/products/reagents/western-blotting-and-molecular-reagents/purified-mouse-anti-srebp-2.557037
• Anti-FASN mouse monoclonal antibody (Santa Cruz Biotechnology, Cat# sc-55580)
Reactivity: Human; Host species: Mouse; Application: WB/IP/IHC/IF
https://www.scbt.com/p/fatty-acid-synthase-antibody-a-5?gad_source=1&gclid=EAIaIQobChMIoojV5ayziQMV7IKDBx3jXBv9EAAYASAAEgLTB_D_BwE
• Anti-LDLR mouse monoclonal antibody (Santa Cruz Biotechnology, Cat# sc-18823)
Reactivity: Human; Host species: Mouse; Application: WB/IP/IHC/IF
https://www.scbt.com/de/p/ldlr-antibody-c7?srsltid=AfmBOoqd0GsMZisONOaXooTHp3ZEweX2zBDuHTjU80zGSg46EYciP1ue
• Anti-D-DT rabbit polyclonal antibody, generated by R. Bucala and colleagues Bucala lab (Yale)
Reactivity: Human/mouse; Host species: Rabbit; Application: WB
(Merk, M., Zierow, S., Leng, L., Das, R., et al. (2011). The D-dopachrome tautomerase (DDT) gene product is a cytokine and functional homolog of macrophage migration inhibitory factor (MIF). PNAS 108, E577-585; Ma, Y., Su, K.N., Pfau, D., Rao, V.S., et al. (2019). Cardiomyocyte d-dopachrome tautomerase protects against heart failure. JCI;Kim, B.S., Tilstam, P.V., Hwang, S.S., et al. (2017). D-dopachrome tautomerase in adipose tissue inflammation and wound repair. J Cell Mol Med 21, 35-45; Ochi, A., Chen, D., Schulte, W., Leng, etal. (2017). MIF-2/D-DT enhances proximal tubular cell regeneration through SLPI- and ATF4-dependent mechanisms. Am J Physiol Renal Physiol 313, F767-F780).
• Anti-AMPKα rabbit monoclonal antibody (Cell Signaling Technology, Cat# 5832S)
Reactivity: Human/Mouse Rabbit; Host species: Rabbit; Application: WB/IP
https://www.cellsignal.com/products/primary-antibodies/ampka-d63g4-rabbit-mab/5832?srsltid=AfmBOopC3scgKmIbcHCLzO3wkFzWC9PrZ_QSlGiLnsXsY-F-G5SWqGal
• Anti-phospho-AMPKα rabbit monoclonal antibody 1:1000 (Cell Signaling Technology, Cat# 2535)
Reactivity: Human/Mouse Rabbit; Host species: Rabbit; Application: WB/IP/IHC
https://www.cellsignal.com/products/primary-antibodies/phospho-ampka-thr172-40h9-rabbit-mab/2535
• Anti-Akt (pan) (C67E7) Rabbit mAb (Cell Signaling, cat# 4691)
Reactivity: Human/Mouse Rabbit; Host species: Rabbit; Application: WB/IP/IHC/IF/Flow
https://www.cellsignal.com/products/primary-antibodies/akt-pan-c67e7-rabbit-mab/4691?srsltid=AfmBOopvivNK8G39tTGwH4C4htH3TqS69bs46xFM5w76SfgJdw0uteWI
• Anti-phospho-Akt (Ser473) antibody (Cell Signaling, Cat#9271)
Reactivity: Human/Mouse Rabbit; Host species: Rabbit; Application: WB/IP/IF/Flow
https://www.cellsignal.com/products/primary-antibodies/phospho-akt-ser473-antibody/9271?srsltid=AfmBOopLQLj6nP8ZTM-d1PLOqhv2OOfEJ9_OKnTLODBJneNL1E_SdVGj
• Anti-β-actin monoclonal antibody (Santa Cruz Biotechnology, cat: sc-47778)
Reactivity: Human/Mouse; Host species: Mouse; Application: WB/IP/IHC/IF/ELISA
https://www.scbt.com/p/beta-actin-antibody-c4?gad_source=1&gclid=EAIaIQobChMI3PGF0bCziQMVppiDBx0NAhXjEAAYAiAAEgLAx_D_BwE
• Goat anti-mouse HRP-conjugated antibody (Abcam, Cat# ab6789)
Reactivity: Mouse; Host species: Goat; Application: WB/IP/IHC/IF/ELISA/Dot
https://www.abcam.com/en-us/search?facets.categoryType=Secondary+Antibodies&facets.hostSpecies=Goat&facets.targetIsotype=IgG&facets.targetSpecies=Mouse&facets.conjugation=HRP&sorting=relevance&utm_source=google&utm_medium=cpc&gad_source=1&gclid=EAIaIQobChMImq-cw7GziQMV7IKDBx0aKzwfEAAYASAAEgLZ2fD_BwE&gclsrc=aw.ds&productcode=AB6789&view=quickview
• Goat anti-rabbit HRP-conjugated antibody (GE Healthcare Cat# NA934V)

Reactivity: Rabbit; Host species: Donkey; Application: WB
https://www.citeab.com/antibodies/3288289-na934-1ml-amersham-ecl-rabbit-igg-hrp-linked-whole
• Anti-CXCR4 rabbit polyclonal antibody (Invitrogen, Cat# PA3-305)
Reactivity: Human/Mouse; Host species: Rabbit; Application: WB/IP/IHC/IF/Flow
https://www.thermofisher.com/antibody/product/CXCR4-Antibody-Polyclonal/PA3-305
• Anti-CD74 (LN2) mouse monoclonal antibody (Santa Cruz Biotechnology Cat# sc-6262)
Reactivity: Human/Mouse/Rat; Host species: Mouse; Application: WB/IP
https://www.biocompare.com/9776-Antibodies/20596115-CD74-LN-2-AC-Antibody/
• Alexa Fluor 647-conjugated donkey anti-rat IgG (H+L) (Jackson ImmunoResearch Europe Ltd., Cat# 712-606-153)
Reactivity: Human/Mouse/Rat; Host species: Donkey; Application: WB
https://www.jacksonimmuno.com/catalog/products/712-606-153
• Alexa Fluor 488-conjugated goat anti-rat IgG (H+L) (Invitrogen; Cat# A-11006)
Reactivity: Rat; Host species: Goat; Application: ICC/IF/Flow
https://www.thermofisher.com/antibody/product/Goat-anti-Rat-IgG-H-L-Cross-Adsorbed-Secondary-Antibody-Polyclonal/A-11006
• Alexa Fluor 555-conjugated goat anti-rabbit IgG (H+L) (Invitrogen, Cat# A-21429)
Reactivity: Rabbit; Host species: Goat; Application: ICC/IF/Flow
https://www.thermofisher.com/antibody/product/Goat-anti-Rabbit-IgG-H-L-Highly-Cross-Adsorbed-Secondary-Antibody-Polyclonal/A-21429
• Anti-CD68 rat monoclonal antibody 1:100 (Bio-Rad Laboratories, Cat# MCA1957GA)
Reactivity: Mouse; Host species: Rat; Application: IHC/IP/ WB/IF
https://www.bio-rad-antibodies.com/results/go?w=Cat%23%20MCA1957GA&ts=custom&null=
• Cy5-conjugated donkey Anti-Rat IgG (H+L) (Jackson ImmunoResearch Europe Ltd., Cat# 712-175-153)
Reactivity: Rat; Host species: Donkey; Application: WB/IHC
https://www.jacksonimmuno.com/catalog/products/712-175-153
• Mouse PE-conjugated anti-CXCR4 antibody (R&D systems, Cat# FAB21651P)
Reactivity: Mouse; Host species: Rat; Application: Flow
https://www.rndsystems.com/products/mouse-cxcr4-pe-conjugated-antibody-247506_fab21651p
• Rat PE-conjugated anti-mouse IgG2B Antibody (R&D systems, Cat# F0132)
Reactivity: Mouse; Host species: Rat; Application: Flow
https://www.rndsystems.com/products/rat-anti-mouse-igg2b-pe-conjugated-antibody_f0132
• mouse anti-cMyc (9E10) mAb 1:200 (Santa Cruz, cat# sc-40)
Reactivity: Human; Host species: Mouse; Application: WB/IP/IF/IHC/Flow/ELISA
https://www.scbt.com/de/p/c-myc-antibody-9e10?srsltid=AfmBOoqGjO2AF73bh-EhmTbin3tAdsF1Ufjh_HXg2cjtud9yw8x-plIe
• rabbit anti-DYKDDK Tag (D6W5B) mAb 1:200 (anti-FLAG, Cell Signaling Technologies, cat# 14793)
Reactivity: All; Host species: Rabbit; Application: WB/IP/IF/IHC/Flow/Chip
https://www.cellsignal.com/products/primary-antibodies/dykddddk-tag-d6w5b-rabbit-mab-binds-to-same-epitope-as-sigma-aldrich-anti-flag-m2-antibody/14793?srsltid=AfmBOoqeA3Y-Zl9kyBf9KJ-SIUjnqIAtTKrSTwMANOYEnDo0Clgs30GY
• Anti-human CD74 (LN2) (Santa Cruz, Cat# sc-6262)
Reactivity: Human/Mouse/Rat; Host species: Mouse; Application: WB/IP
https://www.biocompare.com/9776-Antibodies/20596115-CD74-LN-2-AC-Antibody/
• IgG1 κ light chain antibody (2B6) (Santa Cruz, Cat# sc-69914)
Reactivity: Mouse; Host species: Rat; Application: WB/ELISA
• V450-conjugated rat anti-mouse CD45 (BD Biosciences Cat# 560501)
Reactivity: Mouse; Host species: Rat; Application: Flow
https://www.bdbiosciences.com/en-us/products/reagents/flow-cytometry-reagents/research-reagents/single-color-antibodies-ruo/v450-rat-anti-mouse-cd45.560501
• FITC-conjugated anti-mouse CD3 (Miltenyi Biotec, Cat# 130-119-758)
Reactivity: Mouse; Host species: Rat; Application: Flow
https://www.miltenyibiotec.com/DE-en/products/cd3e-antibody-anti-mouse-17a2.html#conjugate=fitc:size=30-ug-in-200-ul
• APC/Cy7-conjugated anti-mouse CD19 (BioLegend Cat# 115530)
Reactivity: Mouse; Host species: Rat; Application: Flow
https://www.biolegend.com/de-de/products/apc-cyanine7-anti-mouse-cd19-antibody-3903
• PE-conjugated anti-mouse CD11c (BioLegend, Cat# 117308)
Reactivity: Mouse; Host species: Hamster; Application: Flow
https://www.biolegend.com/de-de/products/pe-anti-mouse-cd11c-antibody-1816
• PE/Cy7-conjugated anti-mouse/human CD11b Antibody (BioLegend, Cat# 101216)
Reactivity: Mouse/Human; Host species: Rat; Application: Flow
https://www.biolegend.com/nl-nl/products/pe-cyanine7-anti-mouse-human-cd11b-antibody-1921
• APC-conjugated anti-mouse Ly-6C (BioLegend, Cat# 128016)
Reactivity: Mouse; Host species: Rat; Application: Flow
https://www.biolegend.com/de-de/products/apc-anti-mouse-ly-6c-antibody-6047
• PerCP-conjugated anti-mouse Ly-6G (BioLegend, Cat# 127654)
Reactivity: Mouse; Host species: Rat; Application: Flow
https://www.biolegend.com/de-de/products/percp-anti-mouse-ly-6g-antibody-13351
• APC/Cy7-cponjugated anti-human CD184 (CXCR4) (Biolegend, Cat# 306527)
Reactivity: Human; Host species: Mouse; Application: Flow
https://www.biolegend.com/de-de/products/apc-cyanine7-anti-human-cd184-cxcr4-antibody-12122
• APC/C7-conjugated mouse anti-human IgG2a, κ Isotype Ctrl (Biolegend, Cat# 400229)
Reactivity: Human; Host species: Mouse; Application: Flow

https://www.biolegend.com/de-de/products/apc-cyanine7-mouse-igg2a-kappa-isotype-ctrl-1923?GroupID=BLG15288
• FITC-conjugated mouse anti-human CD74 (BD Pharmingen, Cat# 555540)
Reactivity: Human; Host species: Mouse; Application: Flow
https://www.bdbiosciences.com/en-no/products/reagents/flow-cytometry-reagents/research-reagents/single-color-antibodies-ruo/fitc-mouse-anti-human-cd74.555540
• FITC-conjugated anti-human IgG2 (isotype control) (BD Pharmingen, Cat# 560952)
Reactivity: Human; Host species: Mouse; Application: Flow
https://www.bdbiosciences.com/en-us/products/reagents/flow-cytometry-reagents/research-reagents/single-color-antibodies-ruo/fitc-mouse-anti-human-igg.560952

Eukaryotic cell lines

Policy information about cell lines and Sex and Gender in Research

Cell line source(s)

Mono-Mac-6 cells DSMZ (ACC 124); HEK293 cells (ACC 305); Huh-7 cells (CLS 300156, Cell Lines Service; HAoECs (Human Aortic Endothelial cells, C-12271, PromoCell); NIH/3T3 cells ACC (DSMZ ACC 59)

Authentication

• Mono-Mac-6 DSMZ (ACC 124)
https://www.dsmz.de/collection/catalogue/details/culture/ACC-124
• HEK293 DSMZ ACC 305
https://www.dsmz.de/collection/catalogue/details/culture/ACC-305
• Huh7 (CLS 300156, from Cell Lines Service (CLS, Eppelheim, Germany)
https://www.cytion.com/HuH7-Cells/300156
• HAoECs (Human Aortic Endothelial cells, C-12271, PromoCell)
https://promocell.com/de_de/human-aortic-endothelial-cells-haoec.html
• NIH/3T3 DSMZ ACC 59
https://www.dsmz.de/collection/catalogue/details/culture/ACC-59

Mycoplasma contamination

Mycoplasma tests (via qPCR) for the cell lines used were conducted by the provider and/or at intervals in the lab and confirmed negative.

Commonly misidentified lines
(See ICLAC register)

No commonly misidentified lines were used.

Palaeontology and Archaeology

Specimen provenance

None

Specimen deposition

None

Dating methods

None

☐ Tick this box to confirm that the raw and calibrated dates are available in the paper or in Supplementary Information.

Ethics oversight

None

Note that full information on the approval of the study protocol must also be provided in the manuscript.

Animals and other research organisms

Policy information about studies involving animals; ARRIVE guidelines recommended for reporting animal research, and Sex and Gender in Research

Laboratory animals

Apoe−/− mice used in the in vivo and ex vivo atherosclerosis experiments were sex- and age-matched C57BL6/J mice (B6.129P2-Apoe/J), all between 8 to 10 weeks old at the start of the atherosclerosis studies. The Apoe−/− mice were initially obtained from Charles River Laboratories in Sulzfeld, Germany, and subsequently backcrossed at the CSD animal facility prior to use.
C57BL/6J wild-type mice were ordered directly from Charles River (strain code 027).
The Mif-2−/−Apoe−/− mouse line was generated by crossing Mif-2+/− and Apoe−/− mice, with the line housed at CSD in Munich, Germany. Genetic experiments were conducted using eight-week-old female and male Mif-2−/−Apoe−/− and Apoe−/− mice.
Cd74−/− mice, with a C57BL/6-J genetic background, were originally bred at Yale University (Shachar et al., Science 1996). All mice used in the experiments were between 8 to 10 weeks of age.

Wild animals

No wild animals were used in the study.

Reporting on sex

Gender (female and male) was considered in the in vivo atherosclerosis experiments at both early and late stages of this study. However, the isolation of B lymphocytes for the in vitro studies did not take gender into account.

Field-collected samples

No field collected samples were used in the study.

Ethics oversight	All mouse experiments were approved by the Animal Care and Use Committee of the local authorities (Regierung von Oberbayern, ROB; Aktenzeichen Az = ROB-55.2Vet-2532.Vet_02-18-40) and performed in accord with the animal protection representative at the Center for Stroke and Dementia Research (CSD).

Note that full information on the approval of the study protocol must also be provided in the manuscript.

Clinical data

Policy information about clinical studies

All manuscripts should comply with the ICMJE guidelines for publication of clinical research and a completed CONSORT checklist must be included with all submissions.

Clinical trial registration	None
Study protocol	None
Data collection	None
Outcomes	None

Dual use research of concern

Policy information about dual use research of concern

Hazards

Could the accidental, deliberate or reckless misuse of agents or technologies generated in the work, or the application of information presented in the manuscript, pose a threat to:

No	Yes	
☒	☐	Public health
☒	☐	National security
☒	☐	Crops and/or livestock
☒	☐	Ecosystems
☒	☐	Any other significant area

Experiments of concern

Does the work involve any of these experiments of concern:

No	Yes	
☒	☐	Demonstrate how to render a vaccine ineffective
☒	☐	Confer resistance to therapeutically useful antibiotics or antiviral agents
☒	☐	Enhance the virulence of a pathogen or render a nonpathogen virulent
☒	☐	Increase transmissibility of a pathogen
☒	☐	Alter the host range of a pathogen
☒	☐	Enable evasion of diagnostic/detection modalities
☒	☐	Enable the weaponization of a biological agent or toxin
☒	☐	Any other potentially harmful combination of experiments and agents

Plants

Seed stocks	*Report on the source of all seed stocks or other plant material used. If applicable, state the seed stock centre and catalogue number. If plant specimens were collected from the field, describe the collection location, date and sampling procedures.*
Novel plant genotypes	*Describe the methods by which all novel plant genotypes were produced. This includes those generated by transgenic approaches, gene editing, chemical/radiation-based mutagenesis and hybridization. For transgenic lines, describe the transformation method, the number of independent lines analyzed and the generation upon which experiments were performed. For gene-edited lines, describe the editor used, the endogenous sequence targeted for editing, the targeting guide RNA sequence (if applicable) and how the editor was applied.*
Authentication	*Describe any authentication procedures for each seed stock used or novel genotype generated. Describe any experiments used to assess the effect of a mutation and, where applicable, how potential secondary effects (e.g. second site T-DNA insertions, mosiacism, off-target gene editing) were examined.*

Flow Cytometry

Plots

Confirm that:

- [x] The axis labels state the marker and fluorochrome used (e.g. CD4-FITC).
- [x] The axis scales are clearly visible. Include numbers along axes only for bottom left plot of group (a 'group' is an analysis of identical markers).
- [x] All plots are contour plots with outliers or pseudocolor plots.
- [x] A numerical value for number of cells or percentage (with statistics) is provided.

Methodology

Sample preparation	Blood samples were collected in tubes containing EDTA buffer, and blood leukocytes and plasma were separated by centrifuging at 300 x g for 10 minutes at 4°C. For leukocyte counts, red blood cells (RBCs) were depleted using an RBC-lysis buffer (BioLegend) at room temperature, followed by washing and suspending leukocytes in PBS with 0.5% BSA. Cells were stained with the following antibodies: V450-conjugated rat anti-mouse CD45, FITC-conjugated anti-mouse CD3, APC/Cy7-conjugated anti-mouse CD19, PE-conjugated anti-mouse CD11c, PE/Cy7-conjugated anti-mouse/human CD11b, APC-conjugated anti-mouse Ly-6C, and PerCP-conjugated anti-mouse Ly-6G.<br>For B lymphocyte isolation, cell suspensions from the spleen and lymph nodes were prepared by mechanical dissociation and passed through a 40-µm cell strainer in PBS containing 2 mM EDTA and 0.5% BSA. CXCR4 internalization mediated by MIF-2 was assessed by staining cells with APC/Cy7-conjugated anti-human CD184 (CXCR4) or APC/Cy7-conjugated mouse anti-human IgG2aκ isotype.<br>In the homing assay, B lymphocytes labeled with the intracellular Cell Tracker Green dye CMFDA (5-chloromethylfluorescein diacetate) were isolated from blood, spleen, lymph nodes, and bone marrow following the same protocol as described above.
Instrument	BD FACSVerse™ flow cytometer
Software	FloJo software 10.0.7 (Treestar)
Cell population abundance	The purity of the isolated monocytes and B cells was assessed by flow cytometry using APC/Cy7-conjugated anti-mouse CD19 antibodies with a purity range of 95–98%, whereas purified monocytes were checked with PE-conjugated anti-CD14 as previously used in our study (Kontos et al., Nat. Commun., 2020).
Gating strategy	Leukocyte subsets were analyzed using FlowJo software. B-cells were identified as CD45+CD19+; T-cells as CD45+CD3+; Monocytes CD45+ Cd11b+, neutrophils as CD45+ Cd11b+ Ly6G+. A figure exemplifying the gating strategy is provided in the Supplementary. The gating strategy applied here is consistent with that used in our prior study (Kontos et al., Nat. Commun., 2020).

- [ ] Tick this box to confirm that a figure exemplifying the gating strategy is provided in the Supplementary Information.

Magnetic resonance imaging

Experimental design

Design type	*Indicate task or resting state; event-related or block design.*
Design specifications	*Specify the number of blocks, trials or experimental units per session and/or subject, and specify the length of each trial or block (if trials are blocked) and interval between trials.*
Behavioral performance measures	*State number and/or type of variables recorded (e.g. correct button press, response time) and what statistics were used to establish that the subjects were performing the task as expected (e.g. mean, range, and/or standard deviation across subjects).*

Acquisition

Imaging type(s)	*Specify: functional, structural, diffusion, perfusion.*
Field strength	*Specify in Tesla*
Sequence & imaging parameters	*Specify the pulse sequence type (gradient echo, spin echo, etc.), imaging type (EPI, spiral, etc.), field of view, matrix size, slice thickness, orientation and TE/TR/flip angle.*
Area of acquisition	*State whether a whole brain scan was used OR define the area of acquisition, describing how the region was determined.*

Diffusion MRI ☐ Used ☐ Not used

Preprocessing

Preprocessing software	*Provide detail on software version and revision number and on specific parameters (model/functions, brain extraction, segmentation, smoothing kernel size, etc.).*
Normalization	*If data were normalized/standardized, describe the approach(es): specify linear or non-linear and define image types used for transformation OR indicate that data were not normalized and explain rationale for lack of normalization.*
Normalization template	*Describe the template used for normalization/transformation, specifying subject space or group standardized space (e.g. original Talairach, MNI305, ICBM152) OR indicate that the data were not normalized.*
Noise and artifact removal	*Describe your procedure(s) for artifact and structured noise removal, specifying motion parameters, tissue signals and physiological signals (heart rate, respiration).*
Volume censoring	*Define your software and/or method and criteria for volume censoring, and state the extent of such censoring.*

Statistical modeling & inference

Model type and settings	*Specify type (mass univariate, multivariate, RSA, predictive, etc.) and describe essential details of the model at the first and second levels (e.g. fixed, random or mixed effects; drift or auto-correlation).*
Effect(s) tested	*Define precise effect in terms of the task or stimulus conditions instead of psychological concepts and indicate whether ANOVA or factorial designs were used.*

Specify type of analysis: ☐ Whole brain ☐ ROI-based ☐ Both

Statistic type for inference (See Eklund et al. 2016)	*Specify voxel-wise or cluster-wise and report all relevant parameters for cluster-wise methods.*
Correction	*Describe the type of correction and how it is obtained for multiple comparisons (e.g. FWE, FDR, permutation or Monte Carlo).*

Models & analysis

n/a	Involved in the study
☐	☐ Functional and/or effective connectivity
☐	☐ Graph analysis
☐	☐ Multivariate modeling or predictive analysis

Functional and/or effective connectivity	*Report the measures of dependence used and the model details (e.g. Pearson correlation, partial correlation, mutual information).*
Graph analysis	*Report the dependent variable and connectivity measure, specifying weighted graph or binarized graph, subject- or group-level, and the global and/or node summaries used (e.g. clustering coefficient, efficiency, etc.).*
Multivariate modeling and predictive analysis	*Specify independent variables, features extraction and dimension reduction, model, training and evaluation metrics.*
